# Nutritional c‐Fos Induction Rewires Hepatic Metabolism and Can Promote Obesity‐Associated Hepatocellular Carcinoma

**DOI:** 10.1002/advs.202509755

**Published:** 2025-09-29

**Authors:** Ao Li, Eduardo H. Gilglioni, Wadsen St‐Pierre‐Wijckmans, Leila Hosseinzadeh, Christelle Veyrat‐Durebex, Sumeet Pal Singh, Roberto Coppari, Latifa Bakiri, Esteban N. Gurzov

**Affiliations:** ^1^ Signal Transduction and Metabolism Laboratory Faculty of Medicine Université libre de Bruxelles Brussels B‐1070 Belgium; ^2^ Department of Cell Physiology and Metabolism University of Geneva Geneva 1211 Switzerland; ^3^ Diabetes Center of the Faculty of Medicine University of Geneva Geneva 1211 Switzerland; ^4^ Regenerative Biology Lab Institut de Recherche Interdisciplinaire en Biologie Humaine et Moléculaire (IRIBHM) Université libre de Bruxelles Brussels B‐1070 Belgium; ^5^ Department of Life Sciences School of Natural Sciences Shiv Nadar Institution of Eminence Delhi 201301 India; ^6^ Department of Laboratory Medicine Medical University of Vienna Vienna 1090 Austria

**Keywords:** c‐Fos, HCC, hepatocytes, MASLD, obesity, steatosis

## Abstract

The transcription factor c‐Fos plays a key role in liver metabolism, stress responses, and carcinogenesis. Here, the role of hepatic c‐Fos in the pathophysiology of metabolic dysfunction‐associated steatotic liver disease and hepatocellular carcinoma (HCC) is investigated. In chow‐fed mice, hepatic c‐Fos is induced by insulin after feeding and suppressed by glucagon during fasting. Adenovirus‐mediated hepatic c‐Fos ectopic expression is sufficient to induce insulin resistance in chow‐fed mice. In models of diet‐induced obesity and inducible hepatocyte‐specific *Fos*‐expressing mice, elevated c‐Fos expression is associated with transcriptomic changes in PPAR signaling and fatty acid metabolism pathways. Mechanistically, ectopic c‐Fos expression enhances glycolysis and activates stress‐related MAPK and insulin‐related PI3K‐Akt signaling, which can contribute to metabolic dysregulation. In HCC, persistent c‐Fos expression correlates with activation of PI3K‐Akt, MAPK, and calcium signaling pathways. Functional studies show that c‐Fos knockdown reduces proliferation and restores apoptotic sensitivity in HCC cells under lipotoxic or endoplasmic reticulum stress conditions. These findings identify c‐Fos as a transcriptional regulator responsive to metabolic and hormonal cues, with potential roles in liver metabolic dysfunction and tumorigenesis.

## Graphical abstract

It is shown that hepatic c‐Fos responds to feeding, altering de novo lipogenesis, glycolysis, and a wide range of steatosis‐associated metabolic signaling pathways. Obesogenic diets cause hepatic c‐Fos overexpression, insulin resistance, and the induction of metabolic reprogramming‐related genes. c‐Fos expression prevents hepatocellular carcinoma cell death triggered by lipotoxicity and endoplasmic‐reticulum stress.

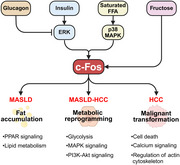

## 1 Introduction

Obesity affects over 1 billion people worldwide and is a major contributor to metabolic‐associated steatotic liver disease (MASLD), which impacts ≈38% of the global population.^[^ ^1^ ^]^ MASLD can progress to severe liver diseases, such as metabolic‐associated steatohepatitis (MASH), cirrhosis, and hepatocellular carcinoma (HCC).^[^ ^2^ ^]^ The liver, as the central hub for metabolic homeostasis, is particularly affected during obesity.^[^ ^3^ ^]^ In recent years, the obesity epidemic has been implicated in up to 40% of the increasing incidence of HCC in developed nations.^[^ ^4^ ^]^

The liver functions by integrating hormonal, nutritional, and neuronal cues to regulate hepatocyte metabolic activity. During fasting, it prioritizes fatty acid oxidation and maintains glucose levels through glycogenolysis and gluconeogenesis, while carbohydrate intake stimulates hepatic glucose uptake, glycogenesis, glycolysis, and lipogenesis.^[^ ^5^, ^6^ ^]^ These metabolic shifts are tightly controlled by insulin, glucagon, and other hormonal cues, adjusting dynamically to nutrient availability and the body's energy needs.^[^ ^6^ ^]^ However, chronic overnutrition and obesity disrupt this finely tuned regulatory network, driving hepatic steatosis, inflammation, and metabolic reprogramming that are key contributors to liver disease progression.^[^ ^2^, ^7^ ^]^

Numerous transcription factors regulate hepatocyte metabolism and contribute to the progression of MASLD, MASH, and HCC. Among them, the activator protein 1 (AP‐1) transcription factor family has been extensively studied for its involvement in cell growth, differentiation, apoptosis, and stress responses, although its specific roles in liver pathology remain incompletely understood.^[^ ^8^, ^9^ ^]^ c‐Fos, the first discovered AP‐1 member, plays a particularly prominent role, orchestrating adaptive metabolic and signaling responses across different cell types.^[^ ^10^ ^]^ c‐Fos expression is a marker of neuronal activation in circuits associated with appetite regulation, including pro‐opiomelanocortin (POMC) neurons^[^ ^11^ ^]^ and corticostriatal circuits,^[^ ^12^ ^]^ both of which influence feeding behavior. In β‐cells, c‐Fos modulates insulin secretion.^[^ ^13^ ^]^ In adipocytes, c‐Fos facilitates adipogenesis by enhancing the expression of key transcription factors such as Peroxisome Proliferator Activated Receptor (PPAR)‐γ.^[^ ^14^ ^]^ In hepatocytes, c‐Fos transcriptionally activates PPARγ and contributes to the development of MASH and HCC in response to palmitate‐rich high‐fat diets.^[^ ^15^, ^16^, ^17^ ^]^ Transcriptomic studies further highlight c‐Fos as a key protein regulating multiple signaling pathways involved in MASLD.^[^ ^18^ ^]^ However, the role of c‐Fos in hepatocyte glucose and lipid metabolism and its contribution to liver dysfunction and disease progression in obesity are still not completely understood.

Here, we studied hepatic c‐Fos expression in chow diet‐fed and obesogenic diet‐fed mice under fasting and feeding conditions. Insulin stimulates c‐Fos expression in hepatocytes, while obesogenic diet components, such as saturated fatty acids and fructose, enhance its expression. Increased c‐Fos alters the expression of genes regulating glucose and lipid metabolism, leading to metabolic changes associated with MASLD and HCC progression. Our findings establish c‐Fos as a key regulator of hepatic energy metabolism and underscore its role in metabolic liver diseases in obesity.

## 2 Results

### 2.1 Hepatic c‐Fos is Induced by Insulin During Feeding

**Figure 1:**
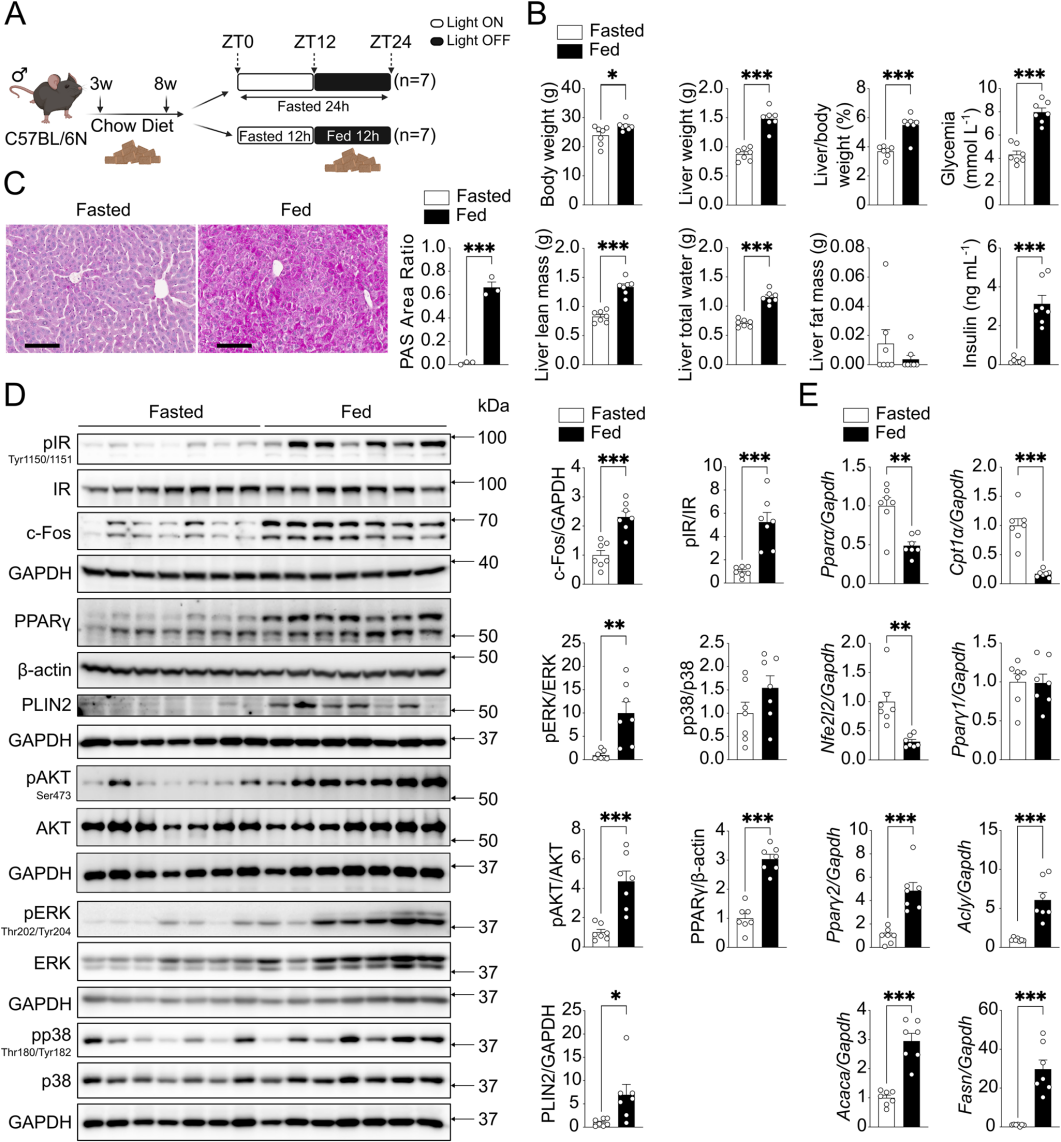
Hepatic c‐Fos expression increases during feeding in chow diet‐fed mice. A) Schematic of the fasted‐fed experimental protocol. Eight‐week‐old C57BL/6N male mice were subjected to either a 24 h fasting protocol (Fasted group: ZT0‐ZT24) or a 12 h fasting (ZT0‐ZT12) followed by 12 h feeding (ZT12‐ZT24) protocol (Fed group). Created in BioRender. Gurzov, E. (2025) https://BioRender.com/j8lyt0y. B) Metabolic differences between fasted and fed groups as indicated (*n* = 7 mice per group). C) Histological analyses using PAS staining demonstrate glycogen accumulation in mouse livers (*n* = 3 mice per group). Scale bar, 100 µm. D) Immunoblot analysis of fasted and fed mouse liver tissues showing pIR, pAKT, pERK, pp38 MAPK, c‐Fos, and lipogenesis markers (PPARγ and PLIN2) protein levels (*n* = 7 mice per group). E) RT‐PCR analysis shows β‐oxidation‐related genes (*Pparα*, *Cpt1α*, *Nfe2l2*) and lipogenesis‐related genes (*Pparγ*, *Acly*, *Acaca*, *Fasn*) in fasted/fed mouse livers (*n* = 7 mice per group). In B‐E), results are shown as mean ± SEM. Statistical analyses using two‐tailed unpaired Student's *t*‐test B‐E). Statistical significance is indicated as ^*^ *p* < 0.05, ^**^ *p* < 0.01, ^***^ *p* < 0.001.

c‐Fos is rapidly induced in response to stimuli and undergoes gradual degradation.^[^ ^19^ ^]^ To explore its fluctuations during fasting and feeding, we first analyzed a publicly available RNA sequencing dataset (GSE200811) derived from the livers of humanized male chimeric mice sampled every 4 h over the circadian cycle (Zeitgeber Time 0, ZT0: lights on; ZT12: lights off).^[^ ^20^ ^]^ *FOS* mRNA levels remained low and stable throughout the light phase (resting/fasting period) but increased at the onset of the dark phase, when mice are active and typically begin feeding (Figure S1A, Supporting Information). We also observed that *PPARG* mRNA expression paralleled and fluctuated with *FOS* expression (Figure S1A, Supporting Information). To directly investigate feeding‐induced c‐Fos expression, 8‐week‐old C57BL/6N lean mice were randomly assigned to one of two experimental conditions: a “24 h‐Fasted” protocol (Fasted group, ZT0 to ZT24) or a “12 h‐Fasted” (ZT0‐12, lights on) & “12 h‐Fed” protocol (Fed group, ZT12‐24, lights off) (**Figure** **1**A). At the end point (ZT24), the fed group exhibited higher body weight, glycemia, and insulinemia when compared to the fasted group (Figure 1B). Liver weight, liver‐to‐body weight ratio, liver lean mass, and liver total water content were also significantly higher in the fed group. No significant differences were observed in liver fat mass. Histological analyses showed larger hepatocytes in the fed group (Figure S1B, Supporting Information), with increased glycogen accumulation (Figure 1C). Increased c‐Fos protein was detected by immunoblotting in the fed group, as well as higher protein expression of the lipogenesis‐associated markers PPARγ and PLIN2 (Figure 1D). In line with increased insulinemia, phosphorylation of the insulin receptor (IR), AKT at Ser473, and ERK1/2 was increased in the fed group. At the mRNA level, β‐oxidation‐related *Pparα* and its downstream gene *Cpt1α*, as well as antioxidant responsive *Nfe2l2*, were decreased in the fed group, while lipogenesis‐related *Pparγ* isoform 2 and its target genes (*Acly*, *Acaca*, and *Fasn*) were significantly upregulated (Figure 1E). The upregulation of lipogenic markers and insulin signaling pathways, along with the downregulation of fatty acid oxidation genes, suggested a shift toward anabolic lipid metabolism in the fed state, which follows the increase in c‐Fos protein.

**Figure 2:**
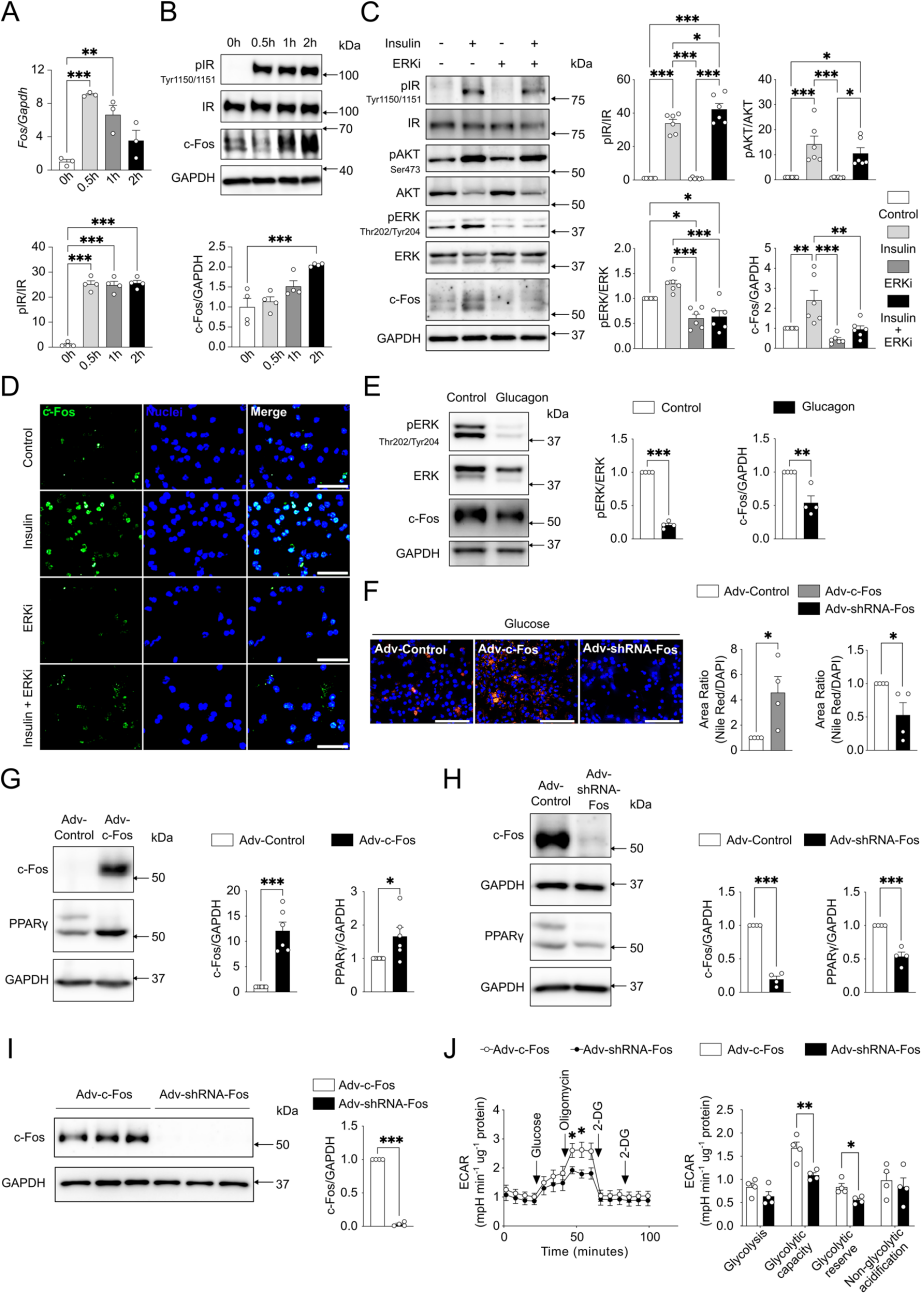
Insulin‐induced c‐Fos expression promotes fat accumulation in primary mouse hepatocytes. A) RT‐PCR (*n* = 3 biological replicates) and B) immunoblot analysis (*n* = 4 biological replicates) indicating *Fos* mRNA and pIR, c‐Fos protein expression in primary mouse hepatocytes (mHep) with 2 h insulin treatment (100 nM). C) Immunoblot analysis of primary mHep (*n* = 6 biological replicates) showing pIR, pAKT, pERK, and c‐Fos protein expression with insulin (100 nM) and an ERK inhibitor (ERKi, SCH772984, 150 nM) treatment after 2 h. D) c‐Fos protein expression in primary mHep with insulin (100 nM) and an ERK inhibitor (ERKi, SCH772984, 150 nM) treatment after 2 h. Scale bar, 100 µm. E) Immunoblot analysis of primary mHep (*n* = 4 biological replicates) showing pERK and c‐Fos protein expression with glucagon (100 nM) treatment after 2 h. F) Nile Red and DAPI staining demonstrate lipid accumulation in primary mHep (*n* = 4 biological replicates) with adenovirus‐mediated c‐Fos overexpression (Adv‐c‐Fos, 50 MOI) or silencing (Adv‐shRNA‐Fos, 50 MOI) in high glucose (22.2 mm) culture conditions for 24 h. Scale bar, 200 µm. G, H) Immunoblot analysis of primary mHep showing c‐Fos and PPARγ protein expression with Adv‐c‐Fos (50 MOI, *n* = 6 biological replicates) or Adv‐shRNA‐Fos (50 MOI, *n* = 4 biological replicates) and high glucose (22.2 mm) treatment after 24 h. I) Immunoblot analysis of primary mHep (*n* = 4 biological replicates) showing c‐Fos protein expression with Adv‐c‐Fos (50 MOI) and Adv‐shRNA‐Fos (50 MOI). J) Real‐time measurement of the extracellular acidification rate (ECAR) in response to glycolytic modulators reveals changes in glycolytic parameters of primary mHep (*n* = 4 biological replicates). Each individual value represents independent hepatocyte preparations from different mice. In A‐C, E‐J), results are shown as mean ± SEM. Statistical analyses using one‐way ANOVA A‐C) or two‐tailed unpaired Student's *t*‐test E‐J). Statistical significance is indicated as ^*^ *p* < 0.05, ^**^ *p* < 0.01, ^***^ *p* < 0.001.

We hypothesized that hepatic c‐Fos expression was induced by insulin during feeding. DESeq2 analysis on the dataset GSE117741^[^ ^21^ ^]^ of hyperinsulinemic‐euglycemic clamped liver transcriptomic revealed that *Fos* expression increased significantly following hyperinsulinemic clamp (HighIns 3 h vs 20 min, Figure S2A, Supporting Information). While transient hypoglycemia at 20 min corresponded to reduced *Fos* levels, consistent with insulin dose‐dependent regulation and glycemic status (Figure S2A, Supporting Information). Next, primary mouse hepatocytes (mHep) were isolated by a two‐step collagenase perfusion method. Insulin treatment of mHep resulted in a significant increase in *Fos* mRNA levels within 30 min (**Figure** **2**A). pIR protein levels were elevated within 30 min, and c‐Fos protein levels significantly increased after 2 h of insulin treatment (Figure 2B). c‐Fos is induced by insulin through the phosphorylation of ERK1/2 and p38 MAPK in several cell types.^[^ ^22^, ^23^ ^]^ Thus, an ERK phosphorylation inhibitor (ERKi, SCH772984) was included with insulin in mHep cultures. pERK and c‐Fos protein levels were significantly reduced in the presence of ERKi compared to insulin after 2 h of treatment (Figure 2C). Immunofluorescence staining further confirmed reduced insulin‐induced c‐Fos expression in the presence of ERKi (Figure 2D). Conversely, glucagon treatment significantly reduced pERK and c‐Fos protein expression in mHep, highlighting the regulation of c‐Fos by ERK under fasting conditions (Figure 2E).

Adenoviral vectors were used to ectopically express (Adv‐c‐Fos) or silence (Adv‐shRNA‐Fos) c‐Fos in mHep (Figure S2B, Supporting Information). Glucose uptake was assessed across a range of glucose concentrations simulating different physiological states of glycemia, including hypoglycemia (1.5 mm), euglycemia (5.5 mm), mild hyperglycemia (11.11 mm), and severe hyperglycemia (22.22 mm). Glucose uptake increased with elevated glucose concentrations (Figure S2C, Supporting Information), and c‐Fos expression further enhanced glucose uptake at 1.5, 5.5, and 11.1 mm glucose. The effect was not observed at 22.2 mm glucose, but c‐Fos still increased hepatocyte lipid accumulation in this setting (Figure 2F). Ectopic c‐Fos expression led to increased PPARγ protein and suppressed *Pparα*, *Cpt1α*, and *Nfe2l2* transcription, while conversely, c‐Fos silencing lowered PPARγ (Figure 2G,H; Figure S2D, Supporting Information). Furthermore, hepatocyte glycolytic capacity and reserve were significantly increased upon ectopic c‐Fos expression compared to c‐Fos silencing (Figure 2I,J). Taken together, c‐Fos is suppressed by glucagon and induced by insulin through ERK signaling pathway in healthy livers and isolated primary mHep. Furthermore, c‐Fos stimulates glucose uptake, glycolysis, and lipid storage in hepatocytes.

### 2.2 Hepatic c‐Fos Silencing Suppresses PPARγ, and c‐Fos Overexpression Induces Insulin Resistance in Chow Diet‐Fed Mice

**Figure 3:**
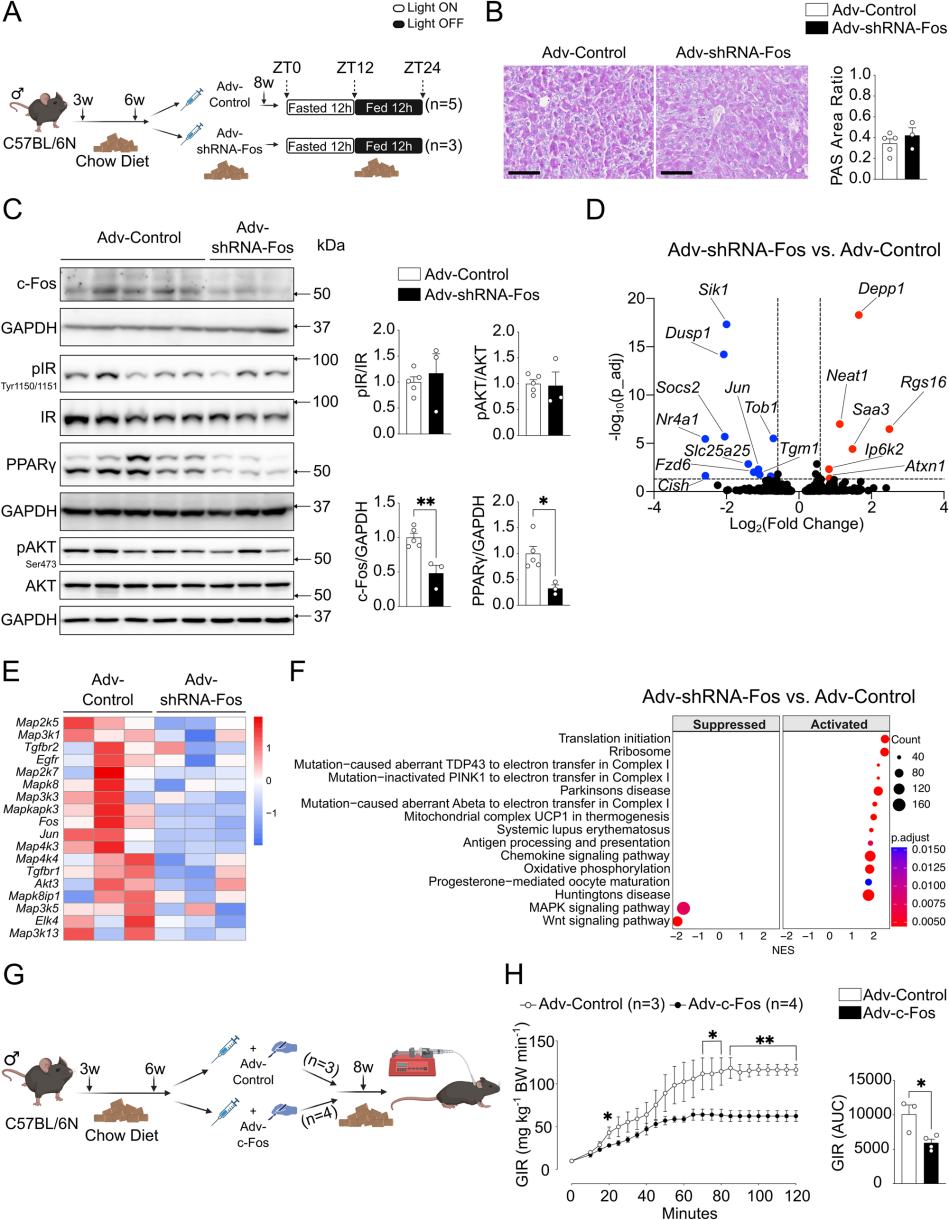
Hepatic c‐Fos silencing suppresses PPARγ, and c‐Fos overexpression induces insulin resistance in chow diet‐fed mice. A) Methodological approach schematic illustrating the fed experimental protocol. Created in BioRender. Gurzov, E. (2025) https://BioRender.com/gqkh5kr. B) Histological analyses using PAS staining demonstrate glycogen accumulation in Adv‐Control (*n* = 5 mice) and Adv‐shRNA‐Fos (*n* = 3 mice) injected mouse livers. Scale bar, 100 µm. C) Immunoblot analysis of Adv‐Control (*n* = 5 mice) and Adv‐shRNA‐Fos (*n* = 3 mice) mouse livers showing pIR, pAKT, c‐Fos, and PPARγ protein expressions. D) RNA‐Seq volcano plot displaying the quantification of transcripts between Adv‐Control (*n* = 3 mice) and Adv‐shRNA‐Fos (*n* = 3 mice) mouse livers. E) RNA‐Seq heatmap displaying alterations in MAPK pathway‐related genes (*n* = 3 mice per group). F) RNA‐Seq KEGG pathway enrichment analysis comparing Adv‐shRNA‐Fos versus Adv‐Control mouse livers (*n* = 3 mice per group). G) Methodological approach schematic illustrating the hyperinsulinemic‐euglycemic clamp experimental protocol. Created in BioRender. Gurzov, E. (2025) https://BioRender.com/ov529kh. H) Glucose infusion rate (GIR) and area under the curve (AUC) between Adv‐Control (*n* = 3 mice) and Adv‐c‐Fos (*n* = 4 mice) as indicated. In B, C, H), results are shown as mean ± SEM. Statistical analyses using two‐tailed unpaired Student's *t*‐test B, C, H). Differential expression analysis using DESeq2 and pathway enrichment analysis using fGSEA with Benjamini‐Hochberg FDR correction D, F). Statistical significance is indicated as ^*^ *p* < 0.05, ^**^ *p* < 0.01.

To validate our findings in vivo, chow diet‐fed 6‐week‐old C57BL/6N mice were randomly assigned to c‐Fos silencing (Adv‐shRNA‐Fos) or control (Adv‐Control) groups (**Figure** **3**A). After 2 weeks, fasted glycemia was measured, and there was no difference between Adv‐shRNA‐Fos and Adv‐Control groups (Figure S3A, Supporting Information). Then, all mice underwent a 12 h fasted & 12 h fed protocol, and the livers were collected at the end point ZT24. No significant changes were detected in body weight, glycemia, hepatic glycogen content, or liver mass and composition (Figure 3B; Figure S3B, Supporting Information). Liver immunoblotting analysis demonstrated efficient c‐Fos silencing and reduced PPARγ protein levels, consistent with the in vitro data (Figure 3C). RNA sequencing was next performed using adenovirus‐infected livers (Figure S3C, Supporting Information). Genes such as the cell cycle regulator *Sik1* and the autophagy regulator *Depp1* exhibited significant alterations in Adv‐shRNA‐Fos livers compared to Adv‐Control, while MAPK signaling tended to be reduced (Figure 3D,E). Gene Set Enrichment Analysis (GSEA)^[^ ^24^, ^25^ ^]^ of Kyoto Encyclopaedia of Genes and Genomes (KEGG) in Adv‐shRNA‐Fos/Adv‐Control revealed several enriched pathways, including suppressed MAPK and Wnt signaling, as well as activated mitochondrial complex UCP1 in thermogenesis and oxidative phosphorylation pathways (Figure 3F). GDF15 is a PPARγ target linked to appetite and weight control in ketogenic diets.^[^ ^26^ ^]^ No significant changes were noted in *Gdf15* expression after c‐Fos knockdown (data not shown), consistent with the lack of change in body weights. This result suggests a context‐ and stage‐dependent regulation of GDF15 by PPARγ.

Next, we ectopically expressed c‐Fos in the liver of chow diet‐fed 6‐week‐old C57BL/6N catheterized mice (Adv‐c‐Fos) for 2 weeks. Mice were subsequently subjected to hyperinsulinemic‐euglycemic clamps to assess insulin sensitivity (Figure 3G). Circulating insulin levels were experimentally increased during the clamp based on body weight (Figure S3D,E, Supporting Information). Decreased insulin sensitivity was observed in Adv‐c‐Fos mice, indicated by a lower glucose infusion rate and a lower glucose area under the curve during clamped euglycemia compared to controls (Adv‐Control) (Figure 3H). Collectively, these data demonstrate that, during feeding, c‐Fos is a key regulator of hepatic PPARγ in the liver and that c‐Fos promotes insulin resistance in chow diet‐fed mice.

### 2.3 Hepatic p38 MAPK and ERK Activation Enhance Feeding‐Induced c‐Fos Expression in Obese Mice

**Figure 4:**
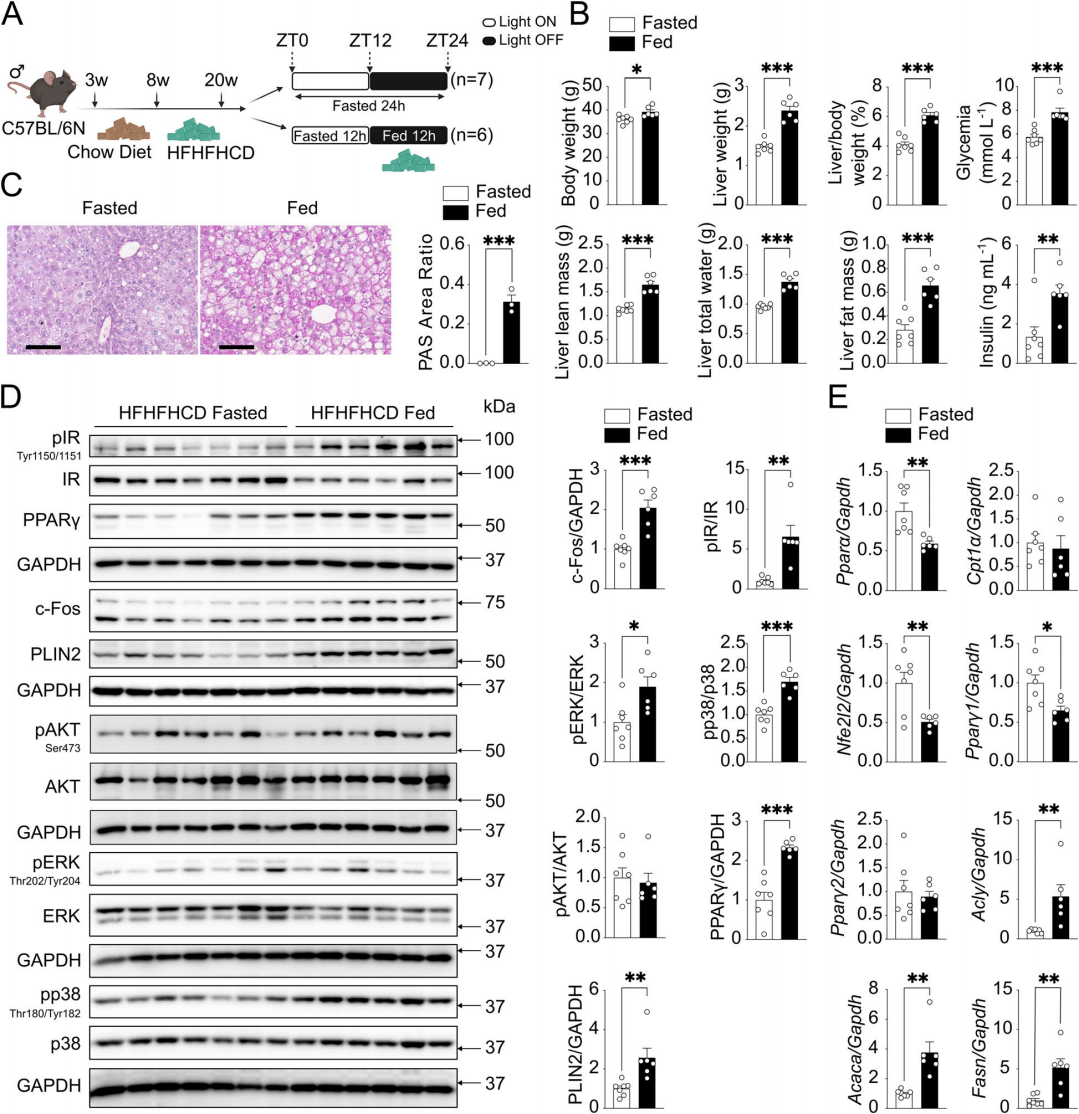
Hepatic c‐Fos expression is enhanced during feeding in HFHFHCD‐fed mice. A) Methodological approach schematic illustrating the fasted‐fed experimental protocol in obese mice fed with 12 weeks of HFHFHCD. Created in BioRender. Gurzov, E. (2025) https://BioRender.com/j8lyt0y. B) Metabolic differences between fasted (*n* = 7 mice) and fed (*n* = 6 mice) groups as indicated. C) Histological analyses using PAS staining demonstrate glycogen accumulation in mouse livers (*n* = 3 mice per group). Scale bar, 100 µm. D) Immunoblot analysis of fasted (*n* = 7 mice) and fed (*n* = 6 mice) mouse liver tissues showing pIR, pAKT, pERK, pp38 MAPK, c‐Fos, and lipogenesis markers (PPARγ and PLIN2) protein expressions. E) RT‐PCR analysis shows β‐oxidation‐related genes (*Pparα*, *Cpt1α*, *Nfe2l2*) and lipogenesis‐related genes (*Pparγ*, *Acly*, *Acaca*, *Fasn*) in fasted (*n* = 7 mice) / fed (*n* = 6 mice) mouse livers. In B‐E), results are shown as mean ± SEM. Statistical analyses using two‐tailed unpaired Student's *t*‐test B‐E). Statistical significance is indicated as ^*^ *p* < 0.05, ^**^ *p* < 0.01, ^***^ *p* < 0.001.

**Figure 5:**
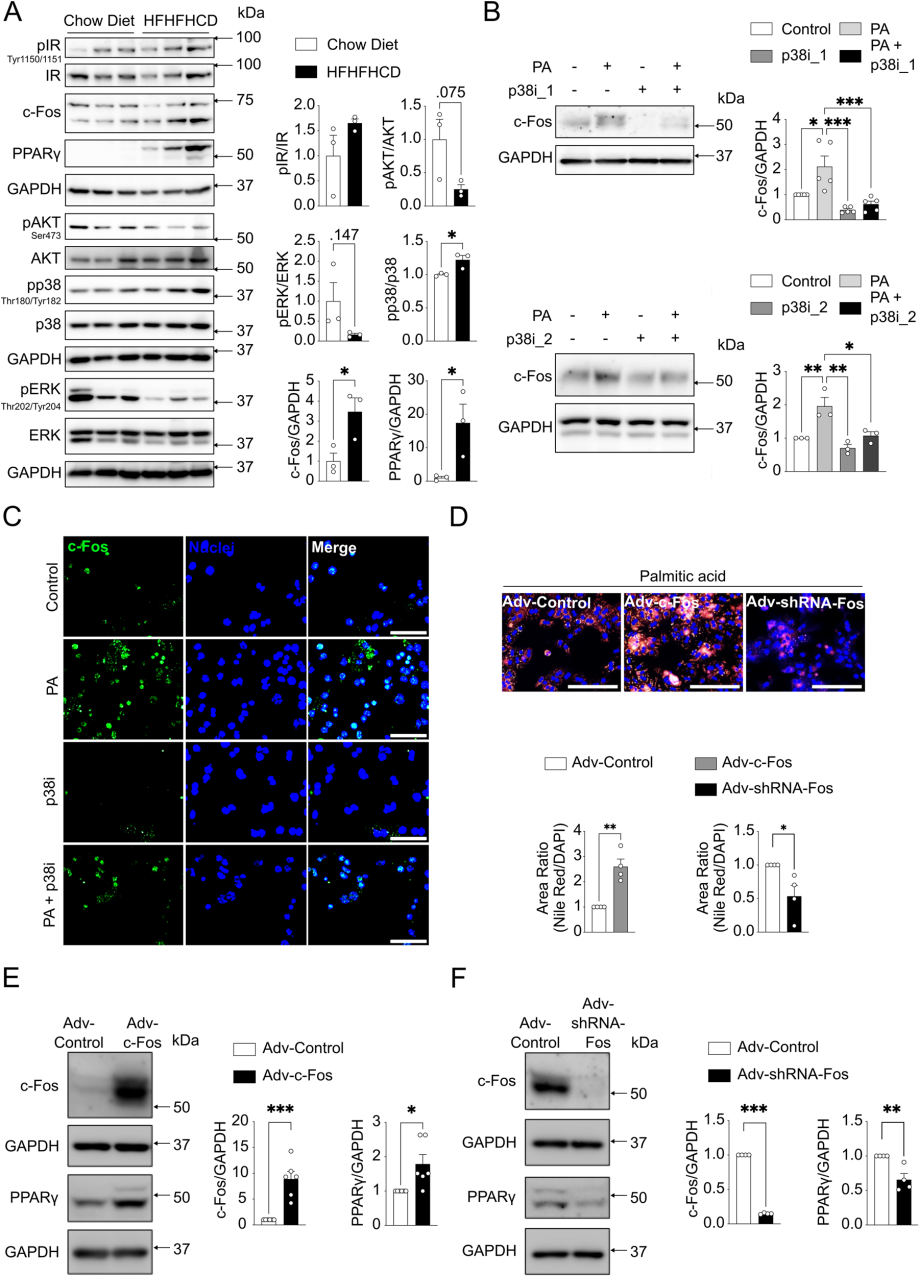
p38 MAPK activation enhances hepatic c‐Fos expression in obese mice. A) Immunoblot analysis of chow diet (*n* = 3 mice) and HFHFHCD (*n* = 3 mice) fed mouse livers showing pIR, pAKT, pERK, pp38 MAPK, c‐Fos, and PPARγ protein expressions. B) Immunoblot analysis of primary mouse hepatocytes (mHep) being treated with palmitic acid (PA, 0.4 mm) and p38 MAPK inhibitor 1 (Adezmapimod, p38i_1, 150 nM, *n* = 5 biological replicates, top) or p38 MAPK inhibitor 2 (Ergothioneine, p38i_2, 250 µm, *n* = 3 biological replicates, bottom) for 4 h, revealing c‐Fos protein expression. C) Immunofluorescence staining demonstrates c‐Fos protein expression in primary mHep with PA (0.4 mm) and p38i_2 (250 µm) treatment after 4 h. Scale bar, 100 µm. D) Nile Red and DAPI staining demonstrate lipid accumulation in primary mHep (*n* = 4 biological replicates) with adenovirus‐mediated control, c‐Fos overexpression (Adv‐c‐Fos, 50 MOI), and silencing (Adv‐shRNA‐Fos, 50 MOI) with 24 h PA (0.1 mm) treatment. Scale bar, 200 µm. E, F) Immunoblot analysis of primary mHep showing c‐Fos and PPARγ protein expression after Adv‐Control, Adv‐c‐Fos (50 MOI, *n* = 6 biological replicates), and Adv‐shRNA‐Fos (50 MOI, *n* = 4 biological replicates) with 24 h PA (0.1 mm) treatment. In A, B, D, E, F), results are shown as mean ± SEM. Statistical analyses using two‐tailed unpaired Student's *t*‐test A, D, E, F) or one‐way ANOVA B). Statistical significance is indicated as ^*^ *p* < 0.05, ^**^ *p* < 0.01, ^***^ *p* < 0.001.

Whether the induction of c‐Fos by feeding in healthy, insulin‐sensitive mice, on a chow diet, is recapitulated in obese mice with steatosis and insulin resistance is not known. Thus, we subjected 8‐week‐old C57BL/6N mice to a high‐fat, high‐fructose, and high‐cholesterol diet (HFHFHCD). After 12 weeks on HFHFHCD, obese mice were randomly assigned to fasted and fed groups, following a protocol similar to the one used for chow‐fed mice (**Figure** **4**A). Obese mice in the fed group exhibited increased body weight, liver weight, liver‐to‐body weight ratio, liver lean mass, liver total water content, glycemia, and serum insulin levels (Figure 4B). In contrast to lean mice, obese mice showed a significant increase in liver fat mass in the fed compared with the fasted condition (Figure 4B). Representative liver H&E and PAS staining revealed larger hepatocytes and increased glycogen accumulation in the fed group (Figure S4A, Supporting Information; Figure 4C). No differences in pAKT levels at Ser473 were observed between fed and fasted groups, confirming insulin resistance in obese mice (Figure 4D). The fed group had elevated levels of pIR, pERK, c‐Fos, PPARγ, and PLIN2, along with significant activation of the stress‐related p38 MAPK. These observations indicate that c‐Fos is induced by HFHFHCD feeding in obese mice and suggest that p38 activation may contribute, together with ERK, to increased c‐Fos expression in this setting. While no differences were observed between fasted and fed groups in *Pparγ2* and *Cpt1α* transcripts, *Pparα and Pparγ1* were downregulated while *Acly*, *Acaca*, and *Fasn* were significantly upregulated in the fed group (Figure 4E). We observed increased PPARγ protein expression but not mRNA levels in the fed state, likely due to the transient timing of transcriptional expression. While post‐translational regulation of PPARγ protein stability cannot be completely excluded, the published evidence^[^ ^16^ ^]^ supports transcriptional regulation as the primary mechanism linking c‐Fos activity to PPARγ expression. We next compared hepatic p38 levels in mice fed with HFHFHCD for 24 weeks with mice on a chow diet (Figure S4B, Supporting Information). Immunoblot analysis showed a significant p38 activation, along with elevated c‐Fos and PPARγ expression in the HFHFHCD‐fed mouse livers, while ERK and AKT activation were unaffected (**Figure** **5**A). Next, isolated mHep were treated with free fatty acids, including palmitic and oleic acid, for 24 h to assess the specific effects of HFHFHCD components. RT‐PCR analysis revealed that palmitic but not oleic acid induced *Fos* mRNA expression (Figure S4C, Supporting Information). p38 MAPK inhibitor prevented palmitic acid‐induced c‐Fos protein expression in mHep (Figure 5B), and this was confirmed by immunofluorescence (Figure 5C). Palmitic acid treatment of mHep pre‐infected with adenovirus‐mediated c‐Fos overexpression led to more lipid droplet accumulation and higher PPARγ expression when compared to mHep with Adv‐Control, and a reduced trend of lipid droplet accumulation and lower PPARγ expression was observed in the Adv‐shRNA‐Fos compared to Adv‐Control (Figure 5D–F). Interestingly, c‐Fos expression was also induced by fructose but not by cholesterol, indicating that not all components of HFHFHCD increase c‐Fos expression in primary mHep (Figure S4D, Supporting Information). However, fructose‐induced c‐Fos could not be suppressed by the p38 inhibitor, suggesting independent mechanisms. Together, these results indicate that saturated free fatty acids and fructose stimulate hepatic c‐Fos expression in the obese mouse liver, at least in part through p38 activation.

### 2.4 c‐Fos Overexpression in Hepatocytes Contributes to MASLD by Modulating Insulin and Stress Signaling Pathways

**Figure 6:**
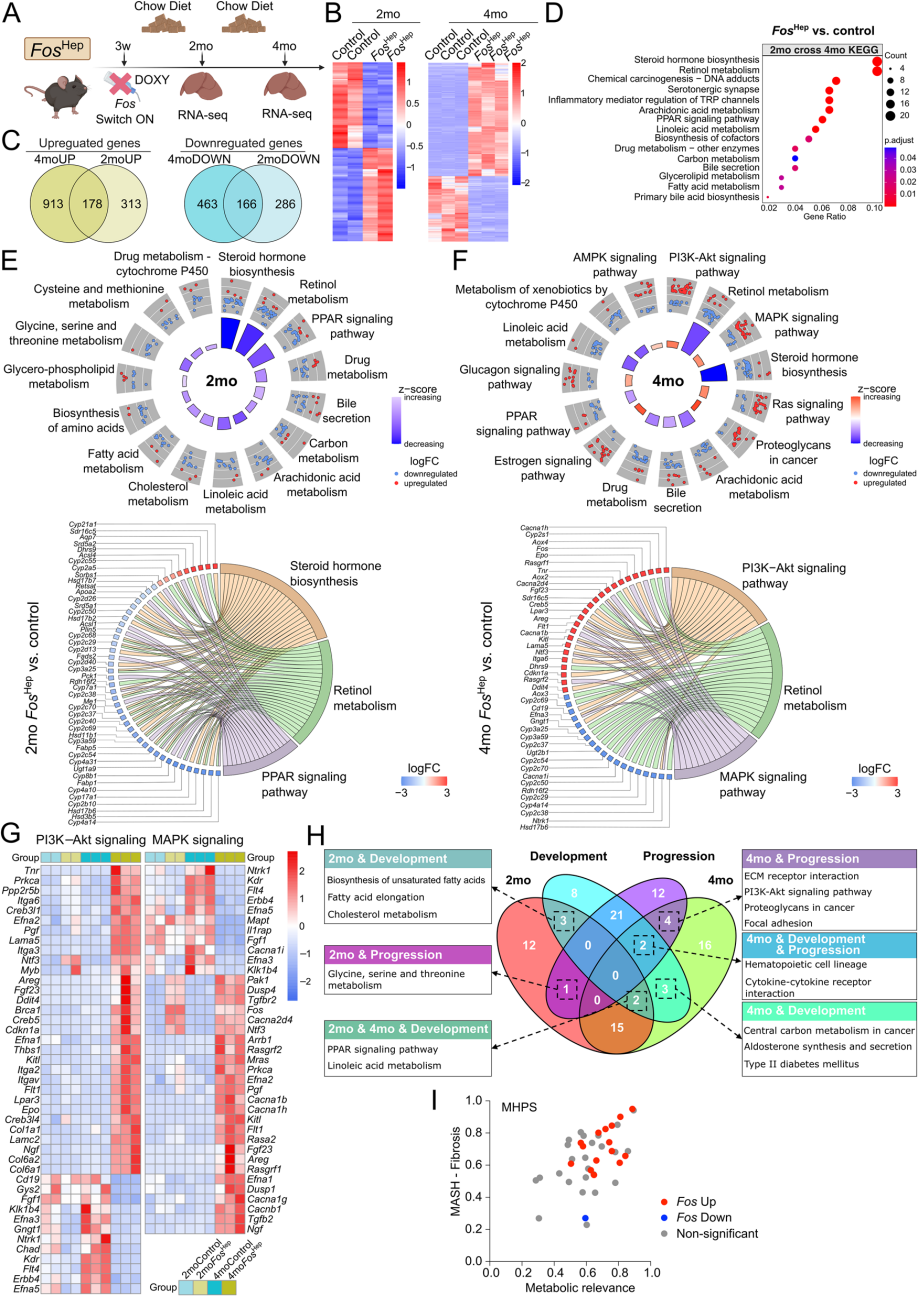
c‐Fos overexpression in hepatocytes contributes to MASLD by modulating insulin and stress signaling pathways. A) Hepatocyte‐specific *Fos* (*Fos* ^Hep^) doxycycline (DOXY)‐inducible mice were subjected to RNA‐Seq (GSE81079) after 2 months (*n* = 2 mice per group) and 4 months (*n* = 3 mice per group) fed with a chow diet. Created in BioRender. Gurzov, E. (2025) https://BioRender.com/temawxv. B) RNA‐Seq heatmaps showing significantly altered genes between control and *Fos* ^Hep^ mice at 2 months (left, *n* = 2 mice per group) and 4 months (right, *n* = 3 mice per group). C) RNA‐Seq Venn diagrams showing the cross‐up‐ (left) and down‐regulated (right) genes between 2 months (*n* = 2 mice per group) and 4 months (*n* = 3 mice per group). D) RNA‐Seq KEGG pathway enrichment analysis using the 2 months (*n* = 2 mice per group) and 4 months (*n* = 3 mice per group) cross‐up‐ and down‐regulated genes from C) as indicated above. E, F) RNA‐Seq circle plots showing the KEGG metabolism‐related pathways in 2 months E) (top, *n* = 2 mice per group) and 4 months F) (top, *n* = 3 mice per group), comparing *Fos* ^Hep^ versus control, respectively. RNA‐Seq chord plots showing top variable genes corresponding to the pathways in 2 months E) (bottom, *n* = 2 mice per group) and 4 months F) (bottom, *n* = 3 mice per group). Genes are ranked by fold change. G) RNA‐Seq heatmap indicating gene alterations in PI3K‐Akt and MAPK signaling pathways between control (2moControl, *n* = 2 mice, 4moControl, *n* = 3 mice) and *Fos* ^Hep^ (2mo*Fos* ^Hep^, *n* = 2 mice, 4mo*Fos* ^Hep^, *n* = 3 mice). H) RNA‐Seq Venn diagram indicating the cross‐pathways among *Fos* ^Hep^/control (2 and 4 months, respectively) and pathways associated with the development and progression stages of MASLD extracted from (PMCID: PMC11199145). I) MHPS (MASLD human proximity score) graph showing the proximity between hepatic c‐Fos upregulation in several rodent models and human MASLD/MASH. Data was extracted from PMCID: PMC11199145. Differential expression analysis using DESeq2 and pathway enrichment analysis using clusterProfiler with Benjamini‐Hochberg B, D, E, F). Z‐SCORE quantification using GOplot E, F).

We next analyzed bulk RNA sequencing datasets generated using a mouse model, where c‐Fos was ectopically expressed in hepatocytes.^[^ ^15^ ^]^ c‐Fos overexpression (*Fos* ^Hep^) was induced on a chow diet at three weeks of age, and the livers were harvested for RNA sequencing analyses 2 and 4 months) later (**Figure** **6**A). In these mice that have a median survival of 5 months, focal liver damage is observed at the 2 months time point, whereas liver dysfunction is molecularly and histologically apparent at 4 months.^[^ ^15^ ^]^ While c‐Fos overexpression in hepatocytes alone does not fully model robust MASLD pathology, transcriptomic analysis from these mice reveals important molecular signatures—including altered PPAR signaling and fatty acid metabolism—that are relevant to steatotic liver disease.^[^ ^15^ ^]^ Importantly, our current study integrates these transcriptomic insights with data from diet‐induced obesity models, in which persistent c‐Fos activation exacerbates MASLD features such as lipid accumulation, insulin resistance, and liver dysfunction. This combination of genetic and metabolic stressors provides a pathophysiologic context on the possible role of c‐Fos in obesity.

Principal Component Analysis (PCA) (Figure S5A, Supporting Information) and heatmaps (Figure 6B) showed significant transcriptional variations between the 2 and 4 months *Fos* ^Hep^ groups compared to their respective controls. A total of 178 upregulated and 166 downregulated mapped genes were shared between the 2 and 4 months groups (Figure 6C). Over Representation Analysis (ORA) of the KEGG revealed commonly altered pathways in 2 and 4 months *Fos* ^Hep^ livers, including PPAR signaling, bile secretion, fatty acid metabolism, and primary bile acid biosynthesis (Figure 6D). Within the PPAR signaling pathway, fold changes in β‐oxidation‐related *Ppara* and *Ppard*, as well as lipogenesis‐related *Pparg* were observed (Figure S5B, Supporting Information). ORA with z‐score^[^ ^27^ ^]^ and GSEA were next applied to evaluate KEGG pathways in the 2 and 4 months groups separately (Figure 6E,F; Figure S5C,D, Supporting Information). Pathways such as steroid hormone biosynthesis, retinol metabolism, PPAR signaling, and bile secretion were downregulated in both *Fos* ^Hep^ groups (Figure 6E, F). Genes of the MAPK signaling pathway were upregulated in both *Fos* ^Hep^ groups, with more pronounced changes observed at 4 months, as identified by both GSEA and ORA with z‐score (Figure 6F; Figure S5C,D, Supporting Information). Additionally, ectopic c‐Fos expression was associated with upregulation of genes in PI3K‐Akt signaling, Ras signaling, proteoglycans in cancer, estrogen signaling, and glucagon signaling (Figure 6F). PI3K‐Akt and MAPK pathways enrichment in *Fos* ^Hep^ livers were highlighted with increased expression of the respective target gene (Figure 6G). Given the wide range of pathways altered by c‐Fos, we compared the 2 and 4 months ORA results with pathways implicated in the development and progression stages of MASLD.^[^ ^18^ ^]^ Overlapping pathways, such as PPAR and PI3K‐Akt signaling, associated with development and progression stages of MASLD and *Fos* ^Hep^ are indicated in Venn diagram (Figure 6H). Additionally, a strong association between hepatic c‐Fos upregulation and liver dysfunction in MASLD/MASH is apparent when mining metabolic relevance and MASH‐Fibrosis scores, replotted from^[^ ^18^ ^]^ (Figure 6I).

Overall, these results suggest that increased hepatic c‐Fos expression promotes MASLD through multiple signaling pathways in the liver, including insulin‐associated PI3K‐Akt and stress‐associated MAPK signaling.

### 2.5 c‐Fos Contributes to Dedifferentiation and Proliferation in HCC Cells

Hepatocyte metabolic reprogramming and inflammation driven by c‐Fos have been linked to liver disease and to the initial steps of HCC in mouse models.^[^ ^15^ ^]^ To examine c‐Fos expression in established HCC, we administered diethyl nitrosamine (DEN) to 2‐week‐old male mice, which were subsequently fed a 37‐week chow diet (Figure S6A, Supporting Information) or a 25‐week HFHFHCD (Figure S6C, Supporting Information), respectively. Hepatic c‐Fos expression positively correlated with insulin receptor phosphorylation in both chow and HFHFHCD‐fed models, and with AKT phosphorylation only in chow‐diet‐fed mice (Figure S6B,D, Supporting Information).

**Figure 7:**
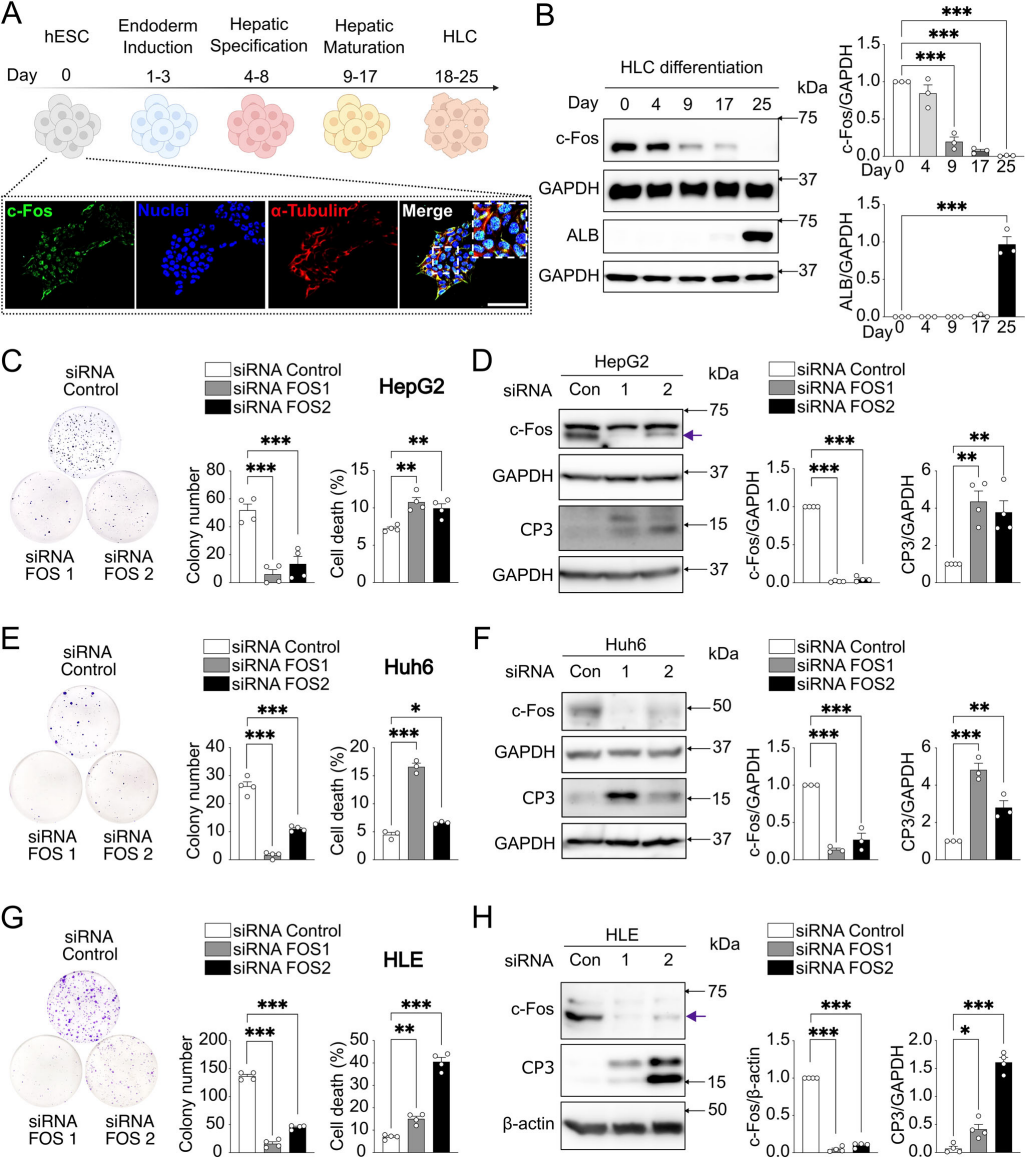
c‐Fos expression reduces susceptibility of HCC cells to apoptosis. A) Methodological approach schematic illustrating hepatocyte‐like cells (HLC) differentiated from H1 human embryonic stem cells (hESC). Immunofluorescence showing c‐Fos nuclear expression in stem cells. Scale bar, 100 µm. Created in BioRender. Gurzov, E. (2025) https://BioRender.com/4f5d1qg. B) Immunoblot analysis of HLC differentiation (*n* = 3 biological replicates) showing c‐Fos and hepatocyte marker ALB protein expression. C, E, G) Colony formation capacity (left) and cell death (right) of HepG2 C) (*n* = 4 biological replicates for both assays), Huh6 E) (*n* = 4 biological replicates for colony formation assay, n = 3 biological replicates for cell death assay), and HLE G) (*n* = 4 biological replicates for both assays) after siRNA‐mediated c‐Fos knockdown as indicated. D, F, H) Immunoblot analysis of HepG2 D) (*n* = 4 biological replicates), Huh6 F) (*n* = 3 biological replicates), and HLE H) (*n* = 4 biological replicates) showing c‐Fos and cleaved caspase 3 (CP3) protein expression after siRNA‐mediated c‐Fos knockdown. Arrow in D, H) indicating c‐Fos. In B‐H), results are shown as mean ± SEM. Statistical analyses using one‐way ANOVA B‐H). Statistical significance is indicated as ^*^ *p* < 0.05, ^**^ *p* < 0.01, ^***^ *p* < 0.001.

During neoplastic transformation to HCC, hepatocytes dedifferentiate and acquire a proliferative phenotype resembling progenitor stem cells. Previous studies demonstrated that c‐Fos gene inactivation facilitates hepatocyte differentiation in both mouse and human liver organoids.^[^ ^28^ ^]^ In a human hepatocyte‐like cell (HLC) differentiation model (**Figure** **7**A), we observed a progressive decrease in c‐Fos expression, while albumin levels significantly increased at the hepatocyte maturation stage (Figure 7B), consistent with previous reports identifying c‐Fos as a suppressor of hepatocyte differentiation.^[^ ^15^, ^28^ ^]^

c‐Fos is expressed across different human HCC cell lines (Figure S7A, Supporting Information). c‐Fos siRNA silencing significantly reduced colony formation in all three cell lines tested, HepG2, Huh6, and HLE (Figure 7C,E,G), accompanied by increased apoptosis, as evidenced by cleaved‐caspase 3 induction (Figure 7D,F,H). c‐Fos has been implicated in promoting tumor invasion associated with JNK/c‐Jun regulation.^[^ ^29^, ^30^, ^31^ ^]^ Colony formation was inhibited at relatively high doses of the JNK inhibitor in HCC cell lines (Figure S7B–D, Supporting Information). These findings suggest that silencing c‐Fos leads to decreased cell proliferation and increased apoptosis in these HCC cell lines, indicating the key role of c‐Fos in survival and proliferation of HCC cells.

### 2.6 c‐Fos Protects HCC Cells from Apoptosis Induced by Lipotoxicity and ER Stress

**Figure 8:**
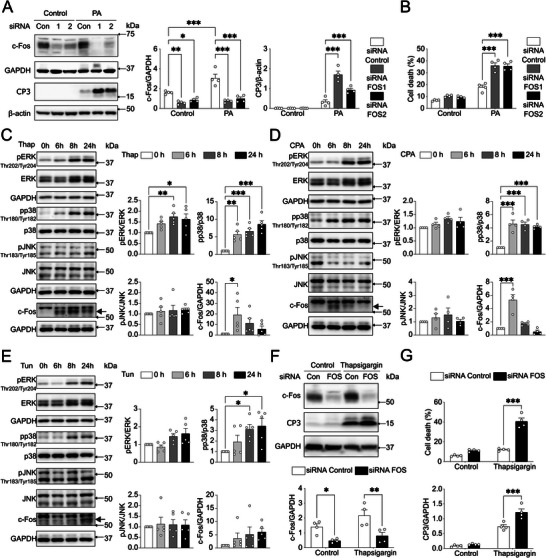
c‐Fos knockdown induces ER stress susceptibility in HCC cells. A, F) Immunoblot analysis of HepG2 cells treated with PA A) (0.5 mm, *n* = 4 biological replicates) or thapsigargin F) (1 µm, *n* = 4 biological replicates) after siRNA‐mediated c‐Fos knockdown, revealing c‐Fos and cleaved caspase 3 (CP3) protein expression. B, G) Cell death analysis of HepG2 cells treated with PA B) (0.5 mm, *n* = 4 biological replicates) or thapsigargin G) (1 µm, *n* = 4 biological replicates) after siRNA‐mediated c‐Fos knockdown. C‐E) Immunoblot analysis of HepG2 cells treated with thapsigargin C) (Thap, *n* = 5 biological replicates, 1 µm), cyclopiazonic acid D) (CPA, *n* = 4 biological replicates, 75 µm), and tunicamycin E) (Tun, *n* = 5 biological replicates, 5 µg mL^−1^), revealing pERK, pJNK, pp38 MAPK, and c‐Fos protein expression. Arrows in C‐E) indicate c‐Fos. In A–G), results are shown as mean ± SEM. Statistical analyses were performed using two‐way A, B, F, G) or one‐way C–E) ANOVA. Statistical significance is indicated as ^*^ *p* < 0.05, ^**^ *p* < 0.01, ^***^ *p* < 0.001.

Saturated free fatty acids induce cell death in hepatocytes,^[^ ^32^ ^]^ but how cancer cells avoid lipotoxicity is unclear. To investigate whether c‐Fos‐mediated signaling counteracts lipotoxicity‐induced apoptosis, we silenced c‐Fos in HepG2 cells, followed by palmitic acid treatment. This combination significantly increased cleaved caspase‐3 (**Figure** **8**A) and cell death rates compared to palmitic acid treatment alone (Figure 8B).

Palmitic acid induces hepatic endoplasmic reticulum (ER) stress and disrupts Ca^2^⁺ signaling.^[^ ^32^, ^33^ ^]^ HepG2 cells were treated with inhibitors (Figure S7E, Supporting Information) targeting the Sarco/ER Ca^2^⁺‐ATPase (SERCA) pump, including thapsigargin (Figure 8C) and cyclopiazonic acid (Figure 8D), as well as tunicamycin (Figure 8E), an inhibitor of N‐acetylglucosamine‐1‐phosphate transferase. c‐Fos protein levels were transiently upregulated after 6 h of thapsigargin or cyclopiazonic acid treatment. However, p38 MAPK activation was consistently elevated across all treatments, while ERK activation was only observed with thapsigargin (Figure 8C–E). Importantly, c‐Fos siRNA silencing in HepG2 cells led to a marked increase in cell death after 24 h of thapsigargin treatment (Figure 8F,G). These results suggest that c‐Fos activation protects HCC cells from apoptosis induced by lipotoxicity or SERCA‐mediated ER stress.

**Figure 9:**
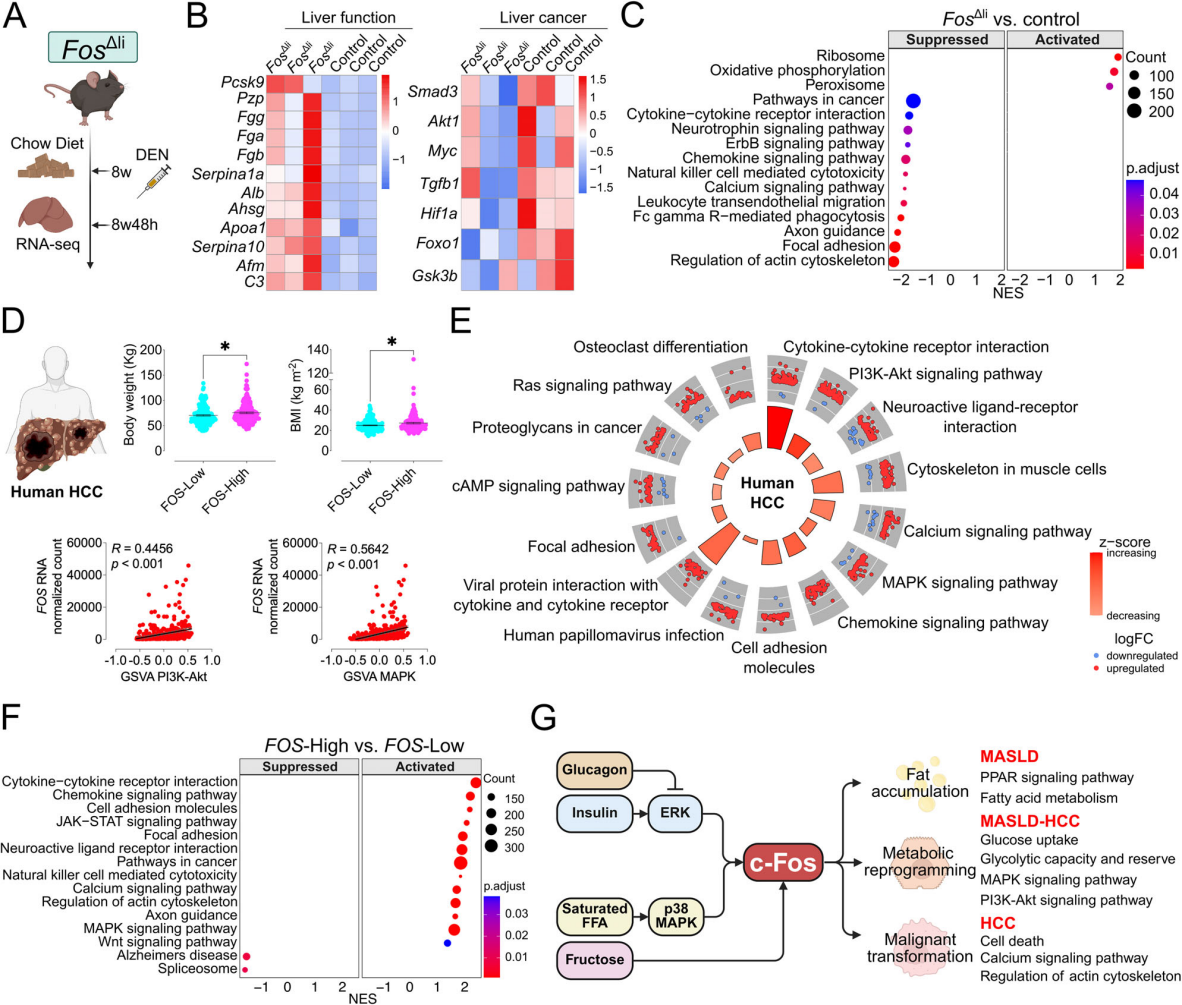
Mouse models with altered *Fos* expression in hepatocytes reveal proximity to human MASLD and the development of HCC. A) Hepatocyte‐specific *Fos* knockout (*Fos* ^Δli^) mice were subjected to RNA‐Seq (GSE81079) after 8 weeks of chow diet and DEN injection for 48 h. Created in BioRender. Gurzov, E. (2025) https://BioRender.com/temawxv. B) RNA‐Seq heatmap displaying liver function‐ and carcinogenesis‐related gene alterations between *Fos* ^Δli^ and control (*n* = 3 mice per group). C) RNA‐Seq KEGG pathway enrichment analysis comparing *Fos* ^Δli^ and control (*n* = 3 mice per group). D) Human HCC data from TCGA were extracted (*n* = 371 patients). *FOS*‐High (*n* = 184 patients) and Low (*n* = 187 patients) groups were previously divided by median *FOS* expression counts in HCC RNA‐Seq before performing DESeq2 analysis. Body weight (*FOS*‐High *n* = 171 patients, *FOS*‐Low *n* = 173 patients) (top left) and BMI (*FOS*‐High *n* = 167 patients, *FOS*‐Low *n* = 168 patients) (top right) as indicated. *FOS* expression level showing a correlation with the customized PI3K‐Akt (bottom left) and MAPK (bottom right) signaling GSVA scores in HCC RNA‐Seq (*n* = 371 patients). Created in BioRender. Gurzov, E. (2025) https://BioRender.com/47vpg9m. E) RNA‐Seq circle plot showing the KEGG pathway enrichment comparing *FOS*‐High (*n* = 184 patients) versus *FOS*‐Low (*n* = 187 patients). F) RNA‐Seq KEGG pathway enrichment analysis comparing *FOS*‐High (*n* = 184 patients) versus *FOS*‐Low (*n* = 187 patients). G) Schematic representation of the postulated c‐Fos mechanism in hepatocytes, illustrating its role in modulating signaling pathways associated with MASLD progression to HCC. https://BioRender.com/zty4uxa. Differential expression analysis was performed using DESeq2 and pathway enrichment analysis using fGSEA with Benjamini‐Hochberg FDR correction C, F). GSVA using a Poisson kernel to estimate the expression distribution of each gene D). Pathway enrichment analysis using clusterProfiler with Benjamini‐Hochberg E). Z‐SCORE quantification was performed using GOplot E). In D), results (top) are shown as mean ± SEM. Statistical analyses were performed using a two‐tailed unpaired Student's *t*‐test D). Correlation analyses were performed using Spearman test D). Data in panels A‐C) were generated using the dataset (GSE81079) from ref. [15] Statistical significance is indicated as ^*^ *p* < 0.05.

To evaluate the impact of c‐Fos deletion in hepatocytes, we analyzed liver RNA‐seq data from a hepatocyte‐specific *Fos* knockout mouse model (*Fos* ^Δli^), previously described by Bakiri et al.^[^ ^15^ ^]^ In this model, Fos is selectively gene‐inactivated in hepatocytes using the *Alfp‐Cre* allele. *Fos* ^Δli^ mice and control (Cre^+^) littermates, which are overall healthy with comparable body and liver weights, were fed a chow diet until 8 weeks‐old, after which they received a single DEN injection. Liver samples were collected 48 h later for RNA sequencing (**Figure** **9**A; Figure S8, Supporting Information). We observed that *Fos* ^Δli^ livers showed a shift toward increased expression of genes involved in oxidative metabolism and decreased expression of genes associated with carcinogenesis (Figure 9B). After performing DESeq2 analysis, GSEA revealed significantly enriched KEGG pathways in *Fos* ^Δli^ livers, including upregulated oxidative phosphorylation and downregulated pathways related to cancer, cytokine‐cytokine receptor interaction, chemokine signaling, calcium signaling, and focal adhesion (Figure 9C). These transcriptomic changes are consistent with the outcome of hepatocyte‐specific c‐Fos deletion in carcinogenesis, functionally demonstrated in Bakiri et al. by injecting *Fos* ^Δli^ mice with DEN at 2 weeks of age and observing greatly diminished liver cancer nodules 9 months later.^[^ ^15^ ^]^

Finally, we investigated c‐Fos‐mediated hepatic signaling alterations in human HCC using RNA sequencing data from The Cancer Genome Atlas (TCGA) Liver Hepatocellular Carcinoma (LIHC) cohort obtained from UCSC Xena^[^ ^34^ ^]^ and analyzed with DESeq2. Genes upregulated in PI3K‐Akt and MAPK signaling pathways from *Fos* ^Hep^ mice (described in Figure 6G) were mapped to their human homologs and assessed using Gene Set Variation Analysis (GSVA) in the TCGA LIHC dataset. GSVA scores for the mouse‐mapped PI3K‐Akt and MAPK pathways positively correlated with *FOS* expression in human tumor tissues (*p* < 0.001) (Figure 9D). Further analysis of human tumor tissues, incorporating clinical data, revealed that patients with relatively high *FOS* hepatic expression also had higher body weight (n = 344) or higher body mass index (BMI) (n = 335, 158/335 BMI > 25 kg m^−^^2^), consistent with increased *FOS* in obesity. ORA with z‐score and GSEA identified several KEGG pathways, including cytokine‐cytokine receptor interaction, calcium signaling, MAPK signaling, cell adhesion molecules, chemokine signaling, and focal adhesion, with upregulated genes in the *FOS*‐High group all associated with malignant hepatocyte transformation (Figure 9E,F). These data further support the validity of our findings using mouse samples and in vitro cell cultures to model human HCC (Figure 9G).

## 3 Discussion

In this study, we elucidated the dynamic regulation of hepatic c‐Fos by nutritional status and hormonal cues, highlighting its dual role in metabolic homeostasis and liver tumorigenesis. Our data reveal that c‐Fos not only promotes metabolic dysregulation under obesogenic conditions but also contributes to oncogenic signaling in hepatocytes, establishing a potential mechanistic link between chronic metabolic stress and HCC development. Our results are consistent with previous work, indicating that dysregulation of lipid metabolism in hepatocytes is intricately tied to transcriptional reprogramming driven by stress‐responsive transcription factors.^[^ ^35^, ^36^ ^]^ Transcriptomic data from c‐Fos‐overexpressing livers^[^ ^15^ ^]^ demonstrate transcriptional programs associated with lipid metabolism and inflammation, hallmarks of MASLD and MASH. However, consistent with prior reports, c‐Fos overexpression alone does not induce overt MASLD phenotypes in chow‐fed mice. In contrast, under diet‐induced obesity conditions, c‐Fos is persistently elevated and acts synergistically with metabolic stress to promote hepatic steatosis, insulin resistance, and inflammatory signaling. These findings support a model in which c‐Fos primes hepatocytes at the transcriptional level, while the pathological progression of MASLD requires additional metabolic stressors. We observed that c‐Fos expression is significantly increased in obese mouse livers, especially during feeding, and that its persistent activation is associated with p38 MAPK signaling. This suggests a metabolic adaptation in obesity where stress‐related pathways compensate for impaired insulin signaling but concurrently drive sustained transcriptional reprogramming that exacerbates lipid accumulation and inflammation, fueling MASLD progression. Our observations align with recent findings by Liang et al., identifying c‐Fos as a negative regulator of hepatocyte maturation in organoid models, where *Fos* knockout promoted hepatocyte differentiation.^[^ ^28^ ^]^ The chronic activation of stress pathways, including c‐Fos, in obesity not only impairs metabolic homeostasis but may also create a pro‐tumorigenic environment through persistent metabolic and inflammatory signaling. These findings are further supported by our data showing that c‐Fos upregulation correlates with increased lipogenesis and insulin resistance, as indicated by elevated PPARγ levels and reduced glucose infusion rates during a hyperinsulinemic‐euglycemic clamp. This effect is likely mediated at the transcriptional level, consistent with prior work showing direct regulation of *Pparg* by AP‐1/c‐Fos dimers.^[^ ^16^ ^]^ While we cannot fully exclude post‐translational mechanisms, the rapid decrease in PPARγ upon c‐Fos silencing supports a loss of transcriptional input. Together with our previous work implicating the JNK/c‐Jun/BIM axis in obesity‐associated liver dysfunction,^[^ ^37^ ^]^ our present data suggest that c‐Fos can dimerize with c‐Jun to modulate hepatic metabolism and insulin signaling.^[^ ^37^ ^]^ Given the established roles of AP‐1 transcription factors in inflammation and transformation, it is plausible that c‐Fos–driven insulin resistance and lipotoxicity synergize with oncogenic pathways such as PI3K‐Akt and MAPK, facilitating the transition from steatosis to malignancy. Our findings conceptually align with previous work^[^ ^38^ ^]^ showing that dietary fat enhances glucose metabolism in non‐transformed hepatocytes and cooperates with oncogenic signaling to promote liver tumorigenesis. In that study, increased lipid availability stimulated glycolysis and proliferation, creating a metabolic state favorable for malignant transformation. Similarly, our data show that feeding‐ and insulin‐induced c‐Fos expression promotes glycolytic flux, lipid accumulation, and inflammatory signaling in hepatocytes, potentially bridging metabolic overload with oncogenic transformation. Although our study does not directly model tumor initiation, it provides mechanistic evidence that c‐Fos acts as a nutrient‐responsive transcriptional regulator that reprograms hepatocyte metabolism toward a tumor‐permissive state in the context of metabolic stress and lipid excess. In line with this, public transcriptomic data from insulin‐infused mice^[^ ^21^ ^]^ showed that hepatic *Fos* expression increases significantly under high insulin infusion, but not in the basal (non‐insulin‐stimulated) state. Interestingly, hypoglycemia at early time points suppressed *Fos* expression, suggesting that c‐Fos dynamics are also influenced by glycemic status. These results strengthen our experimental observations and highlight insulin as a key upstream regulator of c‐Fos in vivo. Together, these data reinforce our conclusion that both insulin and nutritional cues regulate c‐Fos expression in the liver.

In the context of HCC, c‐Fos expression is associated with activation of MAPK signaling pathways, critical mediators of tumor cell survival and metabolic reprogramming. Silencing c‐Fos in HCC cell lines reduced cell proliferation and induced apoptosis, suggesting that c‐Fos plays a crucial role in promoting cell survival under stress, particularly in lipotoxic and ER stress environments.

Our data are also in line with previous findings, showing that c‐Fos plays a critical role in liver inflammation, hepatocyte proliferation, DNA damage response activation, and premalignant transformation.^[^ ^15^ ^]^ We show that c‐Fos enhances glycolytic capacity and reserve in hepatocytes, suggesting that its sustained activation under lipotoxic conditions may prime cells for transformation by establishing a metabolic environment conducive to survival and proliferation. Persistent c‐Fos expression in hepatocytes contributes to preneoplastic transformation, as demonstrated in *Fos* ^Hep^ and *Fos* ^Δli^ mice,^[^ ^15^ ^]^ further emphasizing the dual role of c‐Fos/AP‐1 in metabolic regulation and liver carcinogenesis. In addition, c‐Fos appears to influence tumor metabolism by altering lipid biosynthesis and oxidative pathways in hepatic cancer models.^[^ ^15^ ^]^ In conclusion, our study identifies c‐Fos as a central integrator of metabolic and oncogenic signaling in the liver. Persistent c‐Fos activation under conditions of obesity and lipotoxic stress promotes lipid accumulation and insulin resistance while enabling metabolic reprogramming and survival pathways that support hepatocarcinogenesis. These findings suggest a model in which chronic metabolic dysfunction and c‐Fos activation converge to drive liver pathology across the MASLD‐HCC spectrum, identifying c‐Fos as a potential therapeutic target in metabolic liver disease and cancer.

### 3.1 Limitations and Future Directions

Sex and age‐specific differences, unexplored in this study, could influence c‐Fos dynamics, as estrogen is known to modulate lipid metabolism. Although retro‐orbital delivery of adenoviral vectors is a widely accepted and efficient method for achieving liver‐predominant gene expression, we acknowledge that effects stemming from expression in extrahepatic tissues cannot be entirely excluded. Additionally, this study focuses on the role of c‐Fos in hepatocytes, which may overlook the role of c‐Fos in other liver cell types during MASLD‐HCC development. While our findings suggest that c‐Fos contributes to tumor cell survival under metabolic stress, we did not investigate whether hepatocyte‐specific c‐Fos deletion protects against spontaneous or diet‐induced liver cancer under obesogenic conditions. Future studies should address this by combining metabolic stress models with liver‐specific c‐Fos knockout strategies. Furthermore, the development of specific inhibitors or gene‐editing techniques to modulate c‐Fos/AP‐1 activity in liver cells should be explored to assess their therapeutic potential in liver dysfunction and cancer progression.

## 4 Experimental Section

### Mice

The animal protocols were approved by the Commission of Ethics and Animal Welfare (CEBEA), Faculty of Medicine, Université libre de Bruxelles (Protocol No. 732, and 917). C57BL/6N mice were housed in an animal facility at the ambient temperature of 22–25 °C and 40 – 55% humidity, with the circadian of 12 h light & 12 h dark cycle and free access to diet and water, according to Belgian Regulations for Animal Care.

8‐week‐old male mice were assigned to different groups randomly with consideration of unbiased body weight. These mice were fed with either a chow low‐fat diet (RM1 (P) 801151, Special Diets Services, UK) as a control or a high‐fat, high‐fructose, high‐cholesterol diet (HFHFHCD, 40% kcal from fat, 20% kcal from fructose, 2% cholesterol; D09100310i, Research Diets, New Brunswick, NJ, USA).

### Body and Liver Composition Measurement

The lean and fat mass of the mouse body and liver were measured based on nuclear magnetic resonance with the application of the EchoMRI 3‐in‐1 body composition analyzer from EchoMedical Systems (Houston, TX, USA).

### DEN‐Induced HCC Mouse Model

2‐week‐old male mice were subjected to 1‐time intraperitoneal injection into the lower abdomen with a 25 mg kg^−1^ DEN (dissolved in PBS) to establish a liver tumor model. The mice were maintained on a chow diet until 6 weeks old. Later, the mice were fed either a chow diet until 40 weeks old or an HFHFHCD until 31 weeks old, respectively. Tumor tissue and adjacent non‐tumor tissue were collected after euthanizing the mice by cervical dislocation.

### Histological Analysis

After euthanizing, the mouse liver tissues were collected, dissected, and rinsed with PBS. Then, the tissues were fixed in 4% PFA (pH 7.4), embedded in paraffin, and sectioned into 5–7 µm slices by rotatory microtome, HistoCore MULTICUT R from Leica. After being placed on the positively charged slides, liver sections were stained with hematoxylin and eosin (H&E). Images were captured at 40x magnification by the NanoZoomer Digital Pathology system (Hamamatsu Photonics K.K., version SQ 1.0.9) for analysis.

### Primary Mouse Hepatocyte Isolation, Culture, Treatment, and Staining

Primary mHep were isolated from C57BL/6N mice with a two‐step collagenase perfusion method through the vena cava. After opening the abdominal cavity, the infra‐hepatic vena cava was cannulated for perfusion, and the portal vein was severed to drain the blood from the liver. In the first step, the liver was perfused with HBSS (without calcium and magnesium, Thermo Fisher Scientific, #14170138) supplemented with 10 mm HEPES–NaOH (pH 7.4) and saturated with O2/CO2 (95:5 vol vol^−1^) at 37 °C for 10 min. In the second step, collagenase type IV (0.3 mg mL^−1^) was added to William's E Medium (Thermo Fisher Scientific, #32551087), and perfusion continued for an additional 10 min, softening the liver tissue.

The softened liver was placed in a sterile dish, and the cells were dissociated using a coarse‐toothed comb in cold William's E Medium. The cell suspension was then passed through a 100‐µm filter to remove cell clumps and centrifuged at 50 g for 5 min at 4 °C. The pellet was resuspended in William's E Medium, layered onto a Percoll solution (Millipore Sigma, # GE17‐0891‐01), and centrifuged at 1000 RPM for 10 min.

The isolated primary mHep were seeded with attachment medium (William's Medium with Glutamax, 10% FBS, 1% Penicillin‐Streptomycin, and 10 mm HEPES). The 24‐well plate was seeded with 1 00 000 cells in each well. The 8‐well IBDID plate was seeded with 50 000 cells in each well. The 8‐well Seahorse plate was seeded with 10 000 cells in each well. The attachment medium was changed to maintenance medium (William's Medium with Glutamax, 10% FBS, 1% Penicillin‐Streptomycin, 1% Non‐Essential Amino Acids, 10 mm HEPES, and 5 µm Hydrocortisone) 3–4 h after seeding and underwent overnight recovery.

Insulin (100 nM) and/or ERK inhibitor (150 nM) were added in mHep after 8 h starvation media without serum (William's Medium with Glutamax and 1% Penicillin‐Streptomycin). Glucagon (100 nM) was added in mHep after 22 h of starvation media with 1% FBS. The treatment with oleic acid (0.8 mm), palmitic acid (0.4 mm), and p38 MAPK inhibitor 1 (150 nM, Adezmapimod, HY‐10256) or p38 MAPK inhibitor 2 (250 µm, Ergothioneine, HY‐N1914) included 1% FBS and 1% fatty acid‐free BSA in the media.

Immunofluorescent staining was done in an 8‐well IBDID plate. After treatment, primary mHep were rinsed with PBS and fixed with 4% PFA for 15 min at room temperature. After blocking with UltraVision Protein Block (Thermo Scientific, # TA‐060‐PBQ), the primary antibody (Table S1, Supporting Information) was incubated with primary mHep at 4 °C overnight or at room temperature for 2 h. After rinsing the IBDID plate 3 times with PBS, a secondary antibody was added (Table S2, Supporting Information). After 1 h of incubation at room temperature, the IBDID plate was rinsed with PBS 3 times and then mounted with Antifade Mounting Medium with DAPI (VECTASHIELD, #VEC.H‐1200) for further observation under a fluorescent microscope, ZEISS AXIO Observer. D1.

### Adenovirus Transduction

For in vitro experiments, primary mHep were seeded onto a 24‐well plate for glucose uptake measurement and protein extraction, or an 8‐well IBDID plate for lipid accumulation measurement.^[^ ^37^, ^39^ ^]^ Adenovirus‐mediated Adv‐Control (Ad‐CMV‐Null, #1300, Vector Biolabs), Adv‐c‐Fos (Ad‐CMV‐m‐FOS, SKU: ADV‐259549, Vector Biolabs), and Adv‐shRNA‐Fos (Ad‐GFP‐U6‐m‐FOS‐shRNA, #1835, Vector Biolabs) were transduced in primary mHep after overnight seeding. After 24 h of infection, the culture media were replaced by William's Medium with 1.5, 5.5, 11.1, or 22.2 mm glucose for glucose uptake measurement after 24 h, or replaced by William's Medium together with 1% BSA, 1% FBS, and 0.1 mm palmitic acid for lipid accumulation measurement after 24 h. Glucose content in the medium was measured (GlucCell Glucose Monitoring System; Cesco Bioengineering). The primary mHep in the IBDID plate were fixed with 4% PFA for 15 min at room temperature, stained with 5 µg mL^−1^ Nile Red solution for 20 min, and then mounted with Antifade Mounting Medium with DAPI. Fluorescent images were measured using Fiji (Version: 2.14.0/1.54f) software, and the lipid accumulation area was calculated based on the mean area of each channel (Nile Red and DAPI). For in vivo experiments, chow diet‐fed 6‐week‐old C57BL/6N male mice were infected with Adv‐Control or Adv‐shRNA‐Fos by retro‐orbital injection at a dose of 1.0 × 10^9^ PFU in 100 µl PBS.

### Extracellular Acidification Rates Measurement During Glycolytic Stress Test

Primary mHep were seeded in an 8‐well Seahorse plate. After overnight recovery, the hepatocytes were transduced with Adv‐c‐Fos or Adv‐shRNA‐Fos for 24 h in triplicate wells. Before the start of extracellular acidification rates (ECAR) measurement, the hepatocytes were equilibrated in XF DMEM Medium (5 mm HEPES, glucose‐ and pyruvate‐free, #103575‐100, Agilent Technologies) with 2 mm glutamine in a 37 °C CO_2_‐free incubator for 1 h. Immediately before the ECAR measurement in the glycolytic stress test, the Seahorse plate was refreshed with XF DMEM Medium. During the glycolytic stress test, glucose (10 mm), oligomycin (10 µm), and 2‐DG (in total 100 mm, divided into two consecutive injections of 50 mm) were dissolved in XF DMEM Medium and injected sequentially. The hepatocytes were collected immediately after the glycolytic stress test and stored at −80 °C for protein extraction and immunoblot analysis. The ECAR measurements during the glycolytic stress test were conducted using the XFp Flux Analyzer (Seahorse Bioscience, Agilent Technologies).

### Hyperinsulinemic‐Euglycemic Clamp

Mice were subjected to hyperinsulinemic‐euglycemic clamp as previously described.^[^ ^40^ ^]^ Briefly, 6‐week‐old male mice underwent jugular vein catheterization with exteriorization above the back using a single‐channel vascular access magnetic button (Instech Laboratories, VABM1B/25), and then adenovirus was injected into the jugular vein through the established channel. All the mice were given paracetamol (2 mg ml^−1^) in drinking water for 7 days after the surgery. After 12 days, the mice were subjected to a clamp experiment. During the clamp experiment, the insulin (Novo Nordisk, Actrapid 100 UI ml^−1^) infusion was maintained at 1.5 mIU kg^−1^ body weight min^−1^ to induce hyperinsulinemia. Blood glucose was measured using the StatStrip Xpress2 glucometer (Nova Biomedical, #56506).

### RNA Interference, Colony Formation, and Cell Death Assay

HepG2, HLE, and Huh6 cell lines were cultured in DMEM (Thermo Fisher Scientific, #21885108) supplemented with 10% heat‐inactivated FBS and 1% Penicillin‐Streptomycin. For c‐Fos knockdown, HCC cell lines were transfected with siRNAs targeting c‐Fos or a negative control siRNA (working concentration: 30 nM; QIAGEN, Venlo, the Netherlands). The delivery of siRNA was after 24 h of seeding, facilitated with Lipofectamine RNAiMAX Transfection Reagent (Thermo Fisher Scientific, #13778150) in Opti‐MEM I Reduced Serum Medium (Thermo Fisher Scientific, #31985047) and DMEM with 10% FBS. The siRNA target sequences are provided in Table S3 (Supporting Information). After 24 h of siRNA transfection, HCC cells were recovered for 24 h before trypsinization to obtain a single‐cell suspension. For the colony formation assays, 2000 cells were seeded into P6 plates. Colonies were allowed to grow for 1–2 weeks, depending on the cell lines, and were fixed with 4% PFA and stained with 0.5% crystal violet. In the cell death assay, HCC cell lines recovered for 24 h after siRNA transfection and were treated with/without palmitic acid (0.5 mm) for 24 h. Cell death was assessed using SYTOX green (Thermo Fisher Scientific, Gibco, UK) at a final concentration of 5 µm.

### RNA Extraction, RT‐PCR, and Transcriptomics

The mRNA from primary mHep was extracted with the Dynabeads mRNA DIRECT Purification Kit (Thermo Fisher Scientific, #61012). The total RNA from mouse liver tissues was extracted with the RNeasy Mini Kit (50) (QIAGEN, #74104) and the RNase‐Free DNase Set (50) (QIAGEN, #79254). Reverse transcription from mRNA to cDNA was performed with the Reverse Transcription Core Kit (Eurogentec, #RT‐RTCK‐03). RT‐PCR was conducted on the Bio‐Rad CFX system (Bio‐Rad Laboratories, Hercules, CA) with SsoAdvanced Universal SYBR Green Supermix (Bio‐Rad, #1725274). Primer information is indicated in Table S4 (Supporting Information). The liver total RNA was extracted as described above. The RNA integrity number (RIN) was measured using Agilent 5400. All the RINs were above 9.0. Total RNA quality analysis, library preparation (poly A enrichment), and sequencing were performed by the Novogene (UK) company. NovaSeq X Plus Series (3Gb/sample, 10 M PE reads of PE150) was used for sequencing. Previously published liver RNA‐seq data from DEN‐treated hepatocyte‐specific *Fos*‐knockout mice (*Fos* ^Δli^) were reanalyzed (GSE81079).^[^ ^15^ ^]^ This model was generated by crossing *Fos^f/f ^* mice with *Alfp‐Cre* transgenic mice, resulting in hepatocyte‐specific deletion of *Fos*. The dataset was reprocessed using updated analytical tools to compare gene expression changes under carcinogen‐induced stress.

### Western Blot

RIPA Buffer (Cell Signaling Technology, #9806S) was used to extract total protein lysates from tissues. Cell Lysis Buffer (Cell Signaling Technology, #9803S) was used for extracting cell protein lysates. Both lysis buffers were supplemented with a Halt protease and phosphatase inhibitor cocktail (Thermo Fisher Scientific, #78442). Protein concentration was measured using a BCA protein assay kit (Thermo Fisher Scientific, #PI23227).

20–50 µg of protein lysate mixed with Laemmli buffer was loaded into the gel. The total amount of protein in each loading was the same in one gel. After electrophoretic separation in polyacrylamide gel, the protein was transferred onto a 0.22 µm nitrocellulose membrane (Bio‐Rad, Hercules, CA, USA, #1620112). After blocking with 5% milk in 0.3% TBST, the primary antibody (detailed in Table S1, Supporting Information) was incubated with the membrane in 0.3% TBST with 5% BSA at 4 °C overnight. A secondary antibody was added after washing the membrane 3 times with 0.3% TBST, including goat anti‐rabbit IgG (Dako Agilent, #P0448), goat anti‐mouse IgG (Dako Agilent, #P0447), and goat anti‐guinea pig IgG (PROGEN Biotechnik, #90001) (detailed in Table S2, Supporting Information). For chemiluminescence detection, immunoreactive bands were visualized under the Amersham ImageQuant 800 Western blot imaging system (Cytiva Life Science, Marlborough, MA, USA).

### Bioinformatic Analysis

The RNA sequencing reads of the mouse liver regarding GSE81079 were gathered in unaligned BAM format. The reads were converted to Fastq format by Picard and then aligned to the mouse reference genome (GRCm38.98). After the procedures of FastQC, fastp, Trimmomatic, HISAT2, SAMtools, and featureCounts, the reads were transformed into a count matrix and underwent differential analysis by DESeq2 in R (4.4.1). The identified MASLD pathways, MASLD human proximity scores, and *Fos* expression data were extracted from the PMCID: PMC11199145 source data. Hepatic *Fos* expression in hyperinsulinemic‐euglycemic clamp was extracted from bulk RNA sequencing count data GSE117741. The human HCC dataset of the Cancer Genome Atlas Program (TCGA) was downloaded from UCSC Xena in aligned counts format and analyzed with differential analysis by DESeq2. KEGG pathway enrichment analysis of ORA (using clusterProfiler) with z‐score (using GOplot), GSEA (using fGSEA), and customized pathway analysis of GSVA (using GSVA) followed the instructions on the corresponding websites. ORA was based on differentially expressed genes identified by DESeq2. GSEA was based on pre‐ranked Wald statistics generated from DESeq2. ORA and GSEA were corrected with Benjamini‐Hochberg. The Poisson kernel was used to estimate the expression distribution in GSVA. The adjusted *p*‐value < 0.05 was applied to differentially expressed genes and significantly enriched pathways.

### Stem Cell Differentiation in Hepatocyte‐like Cells

Following a previously established protocol, stem cells were differentiated into hepatocyte‐like cells.^[^ ^39^ ^]^ Laminin 521‐coated plates (BioLamina, Cat#LN521‐05) were prepared, and human H1 embryonic stem cells were detached and seeded onto the laminin‐coated plates (55000 cells/well in P24). The cells were allowed to reach optimal confluency before initiating differentiation.

### Statistical Analysis

The results were presented as mean ± standard error of the mean (SEM). Comparison between two groups was applied with a two‐tailed unpaired Student's *t*‐test. One‐way ANOVA was employed for comparing more than two groups on a single independent variable. For comparisons between groups divided by two independent variables, two‐way ANOVA was employed. The Spearman test was used for correlation analyses between two parameters. Statistical analyses were conducted using Prism software (V.10, GraphPad Software, Inc., La Jolla, CA, USA). The sample size was predetermined based on variability observed in previous experiments and preliminary data. Statistical significance was defined as ^*^ *p* < 0.05, ^**^ *p* < 0.01, ^***^ *p* < 0.001.

## Conflict of Interest

The authors declare no conflict of interest.

## Supporting information



Supporting Information

## Data Availability

The RNA‐Seq dataset generated during the sequencing procedure was deposited in the Gene Expression Omnibus (GEO) database (GSE297224). Additional data and information can be obtained from the corresponding author.
