# Supplementary material for: Nutritional c‐Fos Induction Rewires Hepatic Metabolism and Can Promote Obesity‐Associated Hepatocellular Carcinoma
Source: Adv Sci (Weinh). 2025 Sep 29;12(47):e09755. doi: 10.1002/advs.202509755 (PMC12713005; doi:10.1002/advs.202509755)
Supplement: Supplementary file 1 — Supporting Information [file ADVS-12-e09755-s001.pdf]

Supporting Information

Nutritional c-Fos Induction Rewires Hepatic Metabolism and Can Promote Obesity-Associated Hepatocellular Carcinoma

Ao Li, Eduardo H. Gilglioni, Wadsen St-Pierre-Wijckmans, Leila Hosseinzadeh, Christelle Veyrat-Durebex, Sumeet P. Singh, Roberto Coppari, Latifa Bakiri and Esteban N. Gurzov

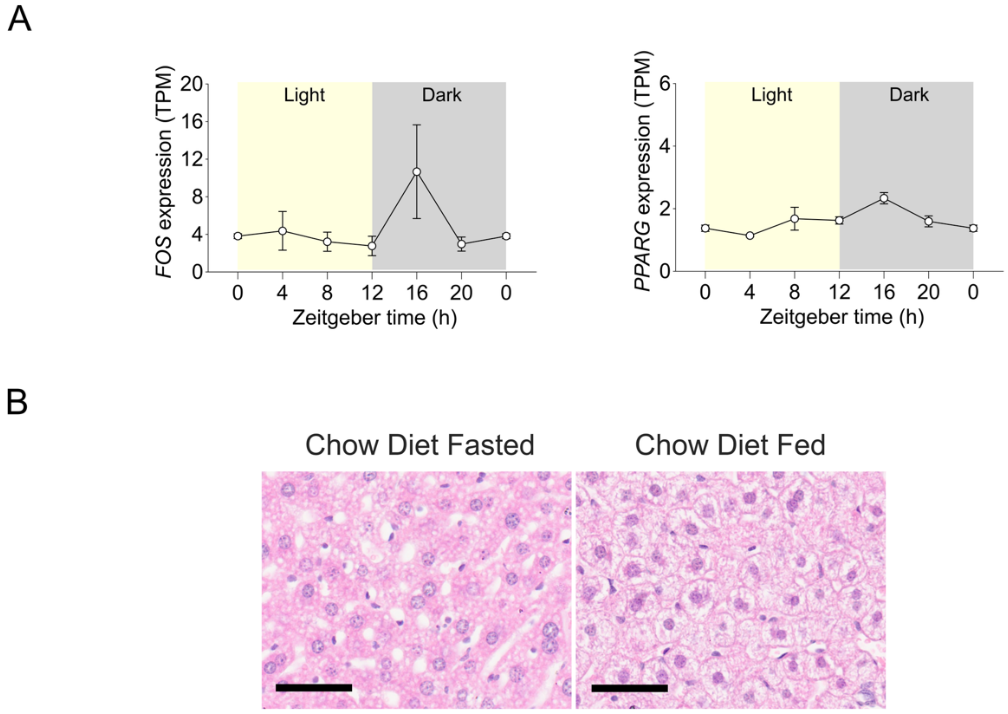

Figure S1. *FOS* expression and hepatocyte structure in fasted/fed livers. A) *FOS* and *PPARG* expression fluctuate within a 24-hour cycle (zeitgeber time) in male humanized liver chimeric mice (PMID: 39209804). *FOS* expression peaks within 4 hours of dark cycle onset and returns to baseline within 8 h, reaching levels comparable to those observed during the light phase (*n* = 3 mice per group). Similarly, *PPARG* expression also peaks within 4 hours after the onset of dark cycle feeding and returns to baseline within 8-12 h, matching levels seen during the light phase (*n* = 3 mice per group). B) Representative H&E staining of mouse livers in fasted and fed groups with a chow diet as indicated. Scale bar, 50µm. In A), results are shown as mean ± SEM.

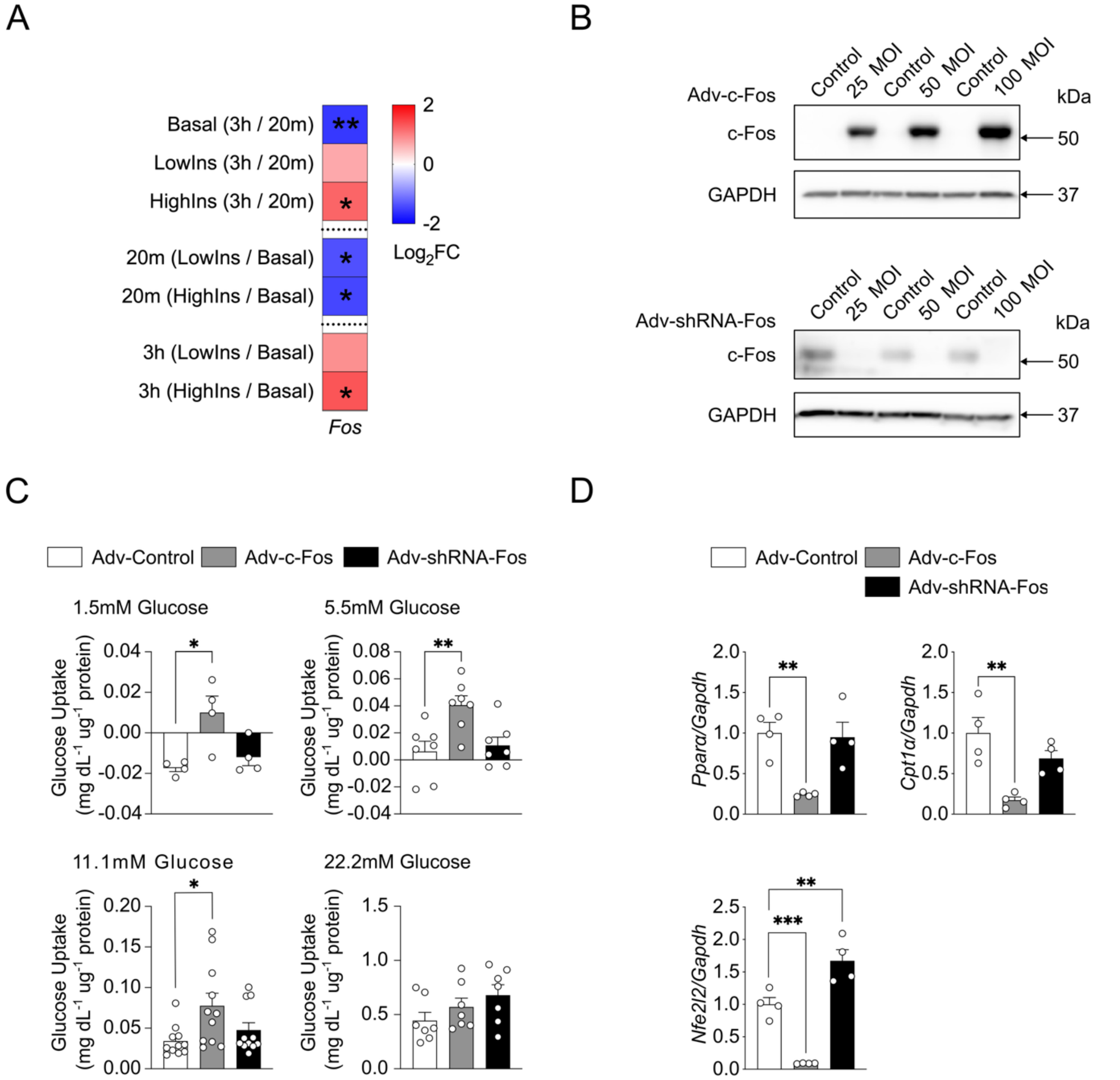

Figure S2. c-Fos enhances glucose uptake and represses oxidative gene programs in hepatocytes and is regulated by insulin *in vivo*. A) Heatmap of liver bulk RNA-seq data (GSE117741), analyzed using DESeq2, showing relative *Fos* expression levels in C57BL/6J male mice 20 min (20 m) and 3 h (3 h) after infusion. Groups included Basal (saline-infused), LowIns (insulin at 4 mU kg^{-1} min^{-1}), and HighIns (insulin at 12 mU kg^{-1} min^{-1}) (n = 5-6 mice per group). B) Representative immunoblot of primary mouse hepatocytes infected with adenoviral vectors to overexpress (Adv-c-Fos, top), silence (Adv-shRNA-Fos, bottom), or control (Adv-Control) for c-Fos expression modulation in different concentrations. 50 MOI was selected to overexpress or silence c-Fos in different experiments in this study. C) Glucose

uptake measurement in primary mouse hepatocytes upon overexpression (50 MOI) and silencing (50 MOI) of c-Fos as indicated. The primary mouse hepatocytes were cultured under 1.5 mM ($n = 4$ biological replicates), 5.5 mM ($n = 7$ biological replicates), 11.1 mM ($n = 11$ biological replicates), and 22.2 mM ($n = 7$ biological replicates) of glucose Williams' E Medium for 24 h. D) RT-PCR analysis revealing β-oxidation related genes *Pparα*, *Cpt1α*, and *Nfe2l2* expression in adenovirus-infected primary mouse hepatocytes with 22.2 mM of glucose Williams' E Medium for 4 h ($n = 4$ biological replicates). In C, D), results are shown as mean ± SEM. Statistical analyses using one-way ANOVA. Statistical significance is indicated as *$p < 0.05$, **$p < 0.01$, ***$p < 0.001$.

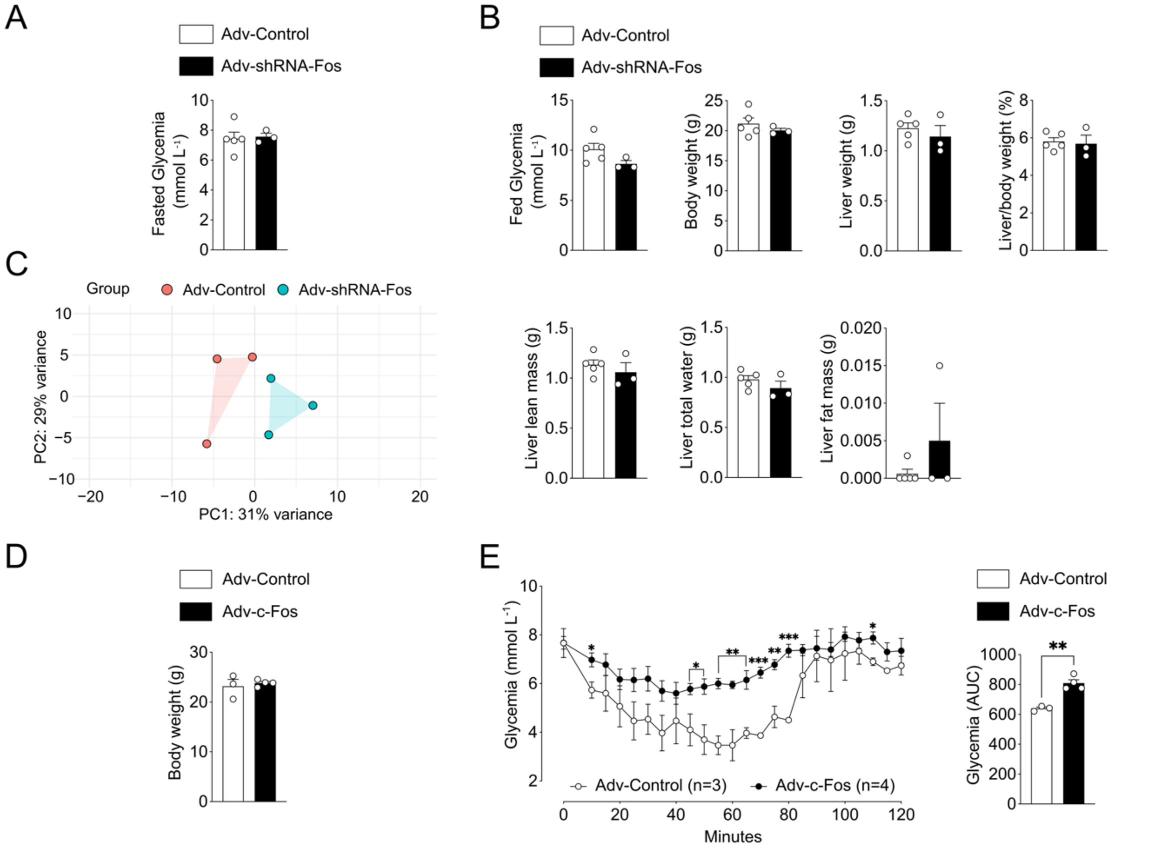

Figure S3. Short-term hepatic c-Fos manipulation alters liver gene expression and body insulin sensitivity in chow diet-fed mice. A) Glycemia of Adv-Control (n = 5 mice) and Adv-shRNA-Fos (n = 3 mice) transduced mice after 5-6 h fasting. B) Metabolic parameters between Adv-Control (n = 5 mice) and Adv-shRNA-Fos (n = 3 mice) transduced mice. C) Principal component analysis (PCA) plot using top 500 variable genes depicting the variation between Adv-Control (n = 3 mice) and Adv-shRNA-Fos (n = 3 mice) liver RNA-Seq data. PC1 and PC2 collectively explained 60 % of the variability between Adv-shRNA-Fos and Adv-Control liver samples, with PC1 accounting for 31 % and PC2 for 29 % of the variance, respectively. D) Body weight between Adv-Control (n = 3 mice) and Adv-c-Fos (n = 4 mice) groups in hyperinsulinemic-euglycemic clamp experiments as indicated. E) Glycemia and area under curve (AUC) between Adv-Control (n = 3 mice) and Adv-c-Fos (n = 4 mice) during clamp as indicated. In A,B,D,E), results are shown as mean ± SEM. Statistical analyses using two-tailed unpaired Student's t-test A,B,D,E). Statistical significance is indicated as *$p < 0.05$, **$p < 0.01$, ***$p < 0.01$.

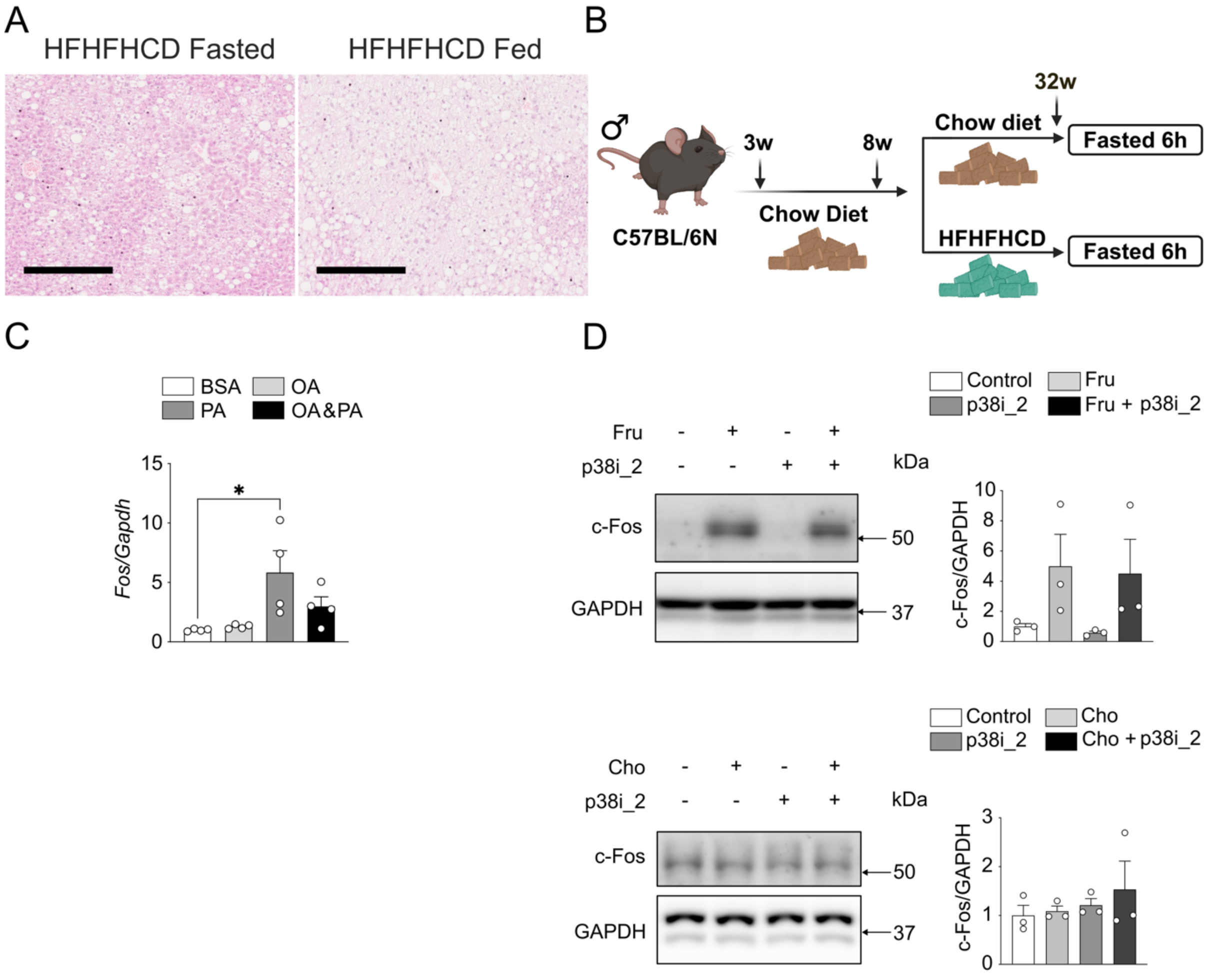

Figure S4. c-Fos is induced by saturated fatty acid palmitic acid and fructose in hepatocytes. A) Representative H&E staining of mouse livers in fasted and fed groups with HFHFHCD diet as indicated. Scale bar, 250µm. B) Methodological approach schematic illustrating the mice fed with 25-week HFHFHCD or a chow diet starting from 8-week to 32-week. Created in BioRender. Gurzov, E. (2025) https://BioRender.com/j8lyt0y. C) RT-PCR analysis shows *Fos* mRNA expression after 24 h of palmitic acid (PA, 0.4 mM), oleic acid (OA, 0.8 mM), or PA&OA treatment in primary mouse hepatocytes (*n* = 4 biological replicates). D) Immunoblot analysis of primary mouse hepatocytes treated with fructose (Fru, 22.2 mM, *n* = 3 biological replicates, top) or cholesterol (Cho, 0.1 µM, *n* = 3 biological replicates, bottom) with p38i_2 (250 µM). In C, D), results are shown as mean ± SEM. Statistical analyses using one-way ANOVA C, D). Statistical significance is indicated as *$p < 0.05$.

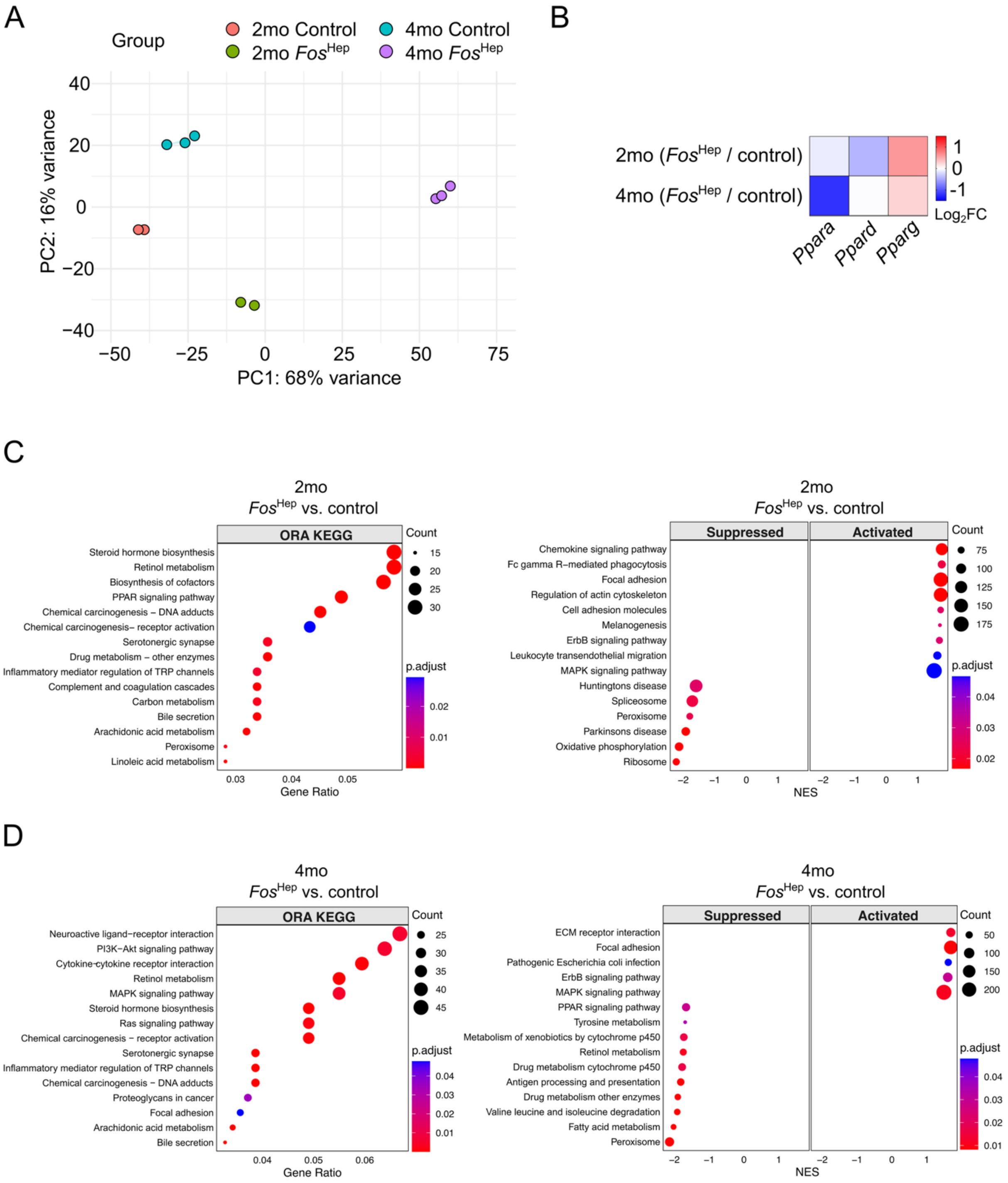

Figure S5. Hepatic c-Fos expression significantly alters KEGG signaling pathways. A) RNA-Seq PCA plot using top 5000 variable genes depicting the variation among 2 months (2mo, control *n* = 2 mice, *Fos*^{Hep} *n* = 2 mice) and 4 months (4mo, control *n* = 3 mice, *Fos*^{Hep} *n* = 3 mice). PC1 and PC2 collectively explained 84% of the variability among different groups, with PC1 accounting for 68 % and PC2 for 16 % of the variance, respectively. B) PPAR genes expression in *Fos*^{Hep}/control mouse livers in 2mo (*n* = 2 mice per group) and 4mo (*n* = 3 mice

per group) as indicated with log_2 fold change. C, D) RNA-Seq KEGG pathway enrichment analysis comparing *Fos*^{Hep} vs. control in 2mo C) (n = 2 mice per group) and 4mo D) (n = 3 mice per group), respectively. KEGG top 15 pathways ranked by gene count in corresponding pathways (ORA left, GSEA right). Differential expression analysis using DESeq2 and pathway enrichment analysis using clusterProfiler (left of C) and D)) and fGSEA (right of C) and D)) with Benjamini-Hochberg.

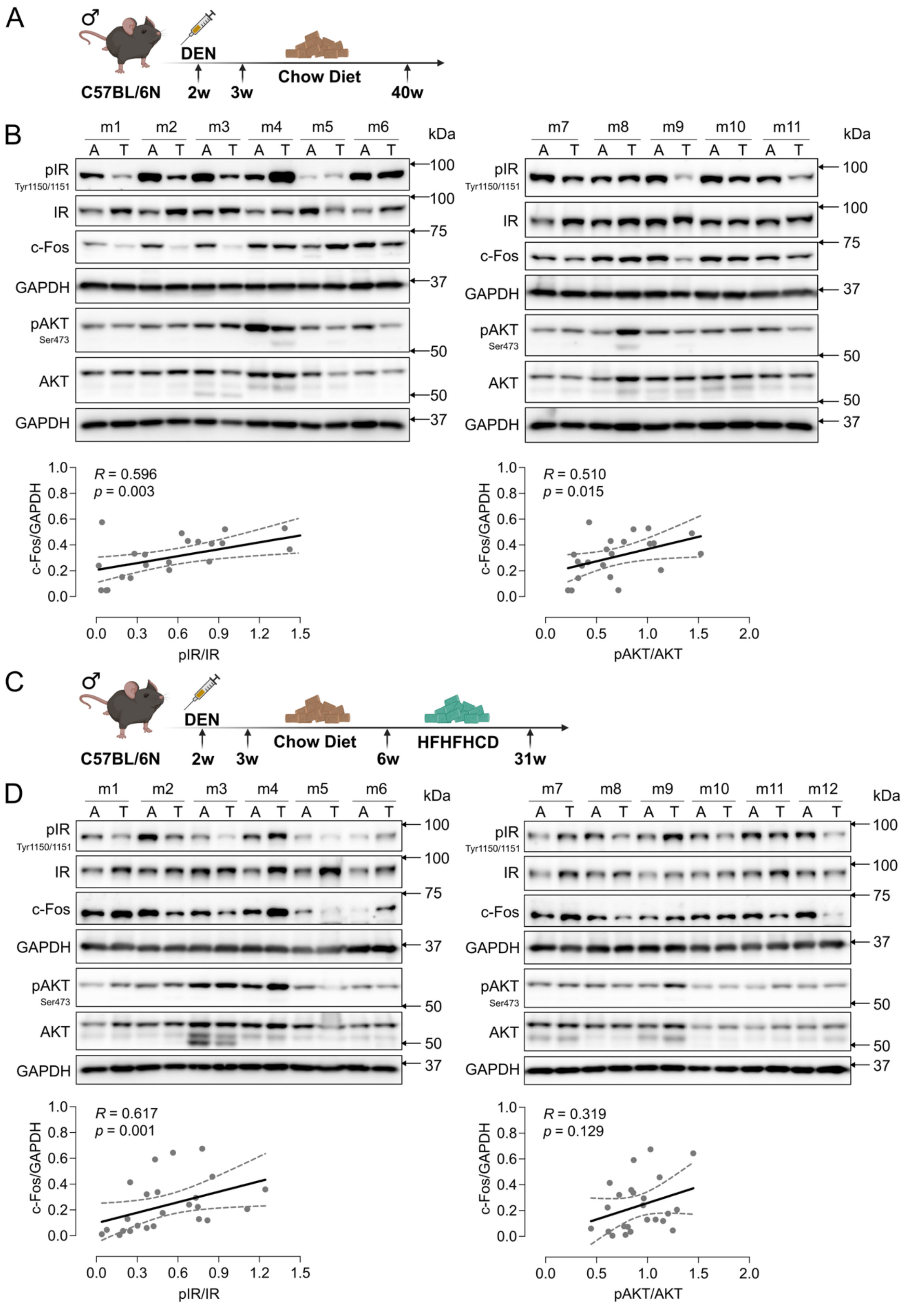

Figure S6. Hepatic c-Fos expression correlates with phosphorylation of insulin receptor in DEN-induced HCC male mouse models. A, C) Methodological approach schematic illustrating diethyl nitrosamine (DEN)-induced HCC male mouse models fed with 37-week

chow diet A) (3-40 weeks duration) or 25-week HFHFHCD C) (6-31 weeks duration) in this study. Created in BioRender. Gurzov, E. (2025) https://BioRender.com/47vpg9m. B, D) Liver adjacent (labelled as A above immunoblot) and tumor tissues (labelled as T above immunoblot) were collected from chow diet B) (n = 11 mice) or HFHFHCD D) (n = 12 mice) fed DEN-induced HCC mice at end points, respectively. Immunoblot analysis of liver adjacent and tumor tissues showing the protein expression levels of pIR, pAKT, and c-Fos. Correlation analyses using Spearman test B, D).

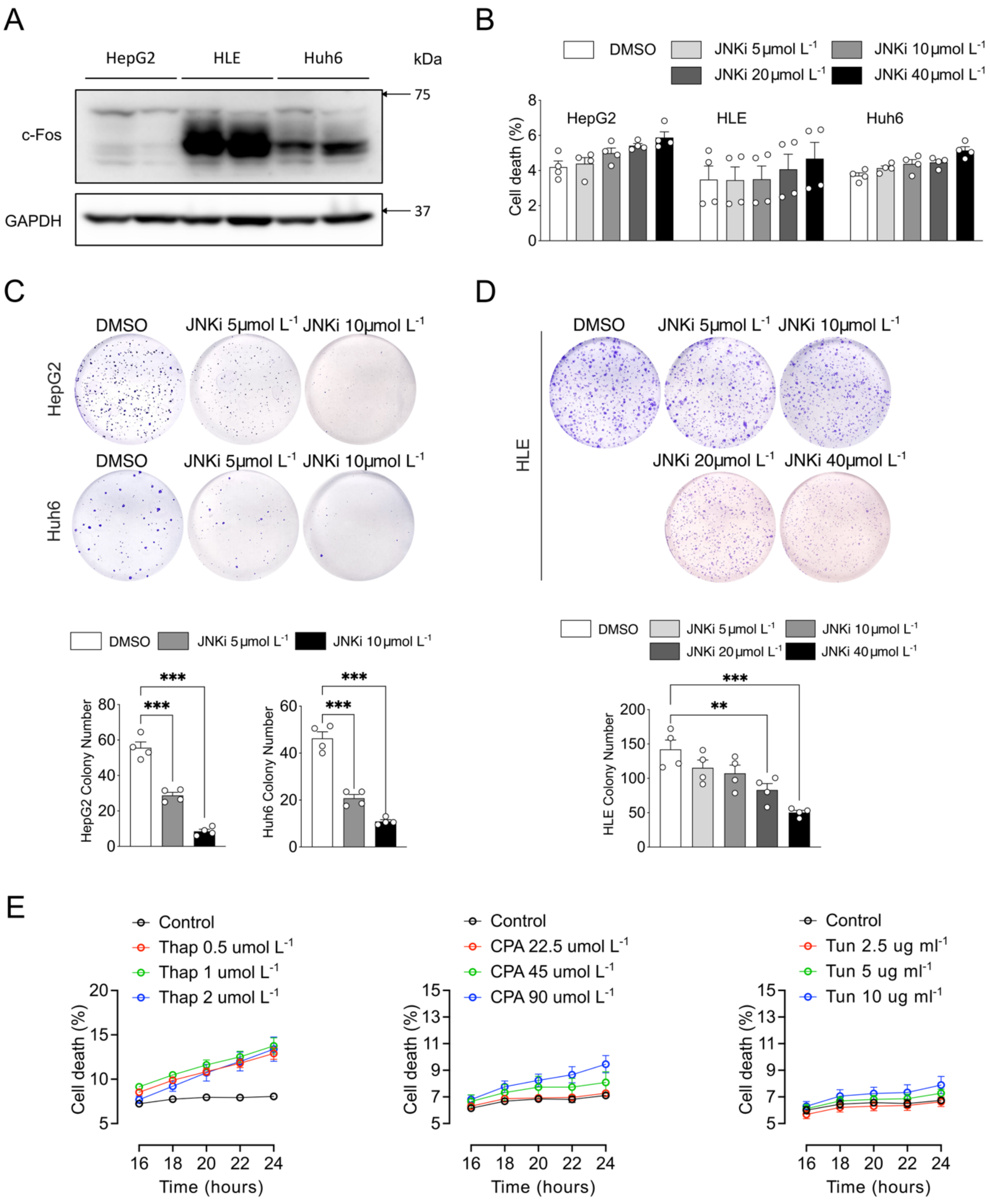

Figure S7. c-Fos expression in HCC cell lines, JNK inhibitor-mediated cell death and colony formation, and ER stressors induced cell death. A) Immunoblot analysis of HepG2, Huh6, and HLE HCC cell lines showing c-Fos protein expression levels (n = 2 biological replicates for each cell line). B) Cell death assay showing different dosages of JNK inhibitor (JNKi) treatment on viability capacity of HepG2, Huh6, and HLE as indicated (n = 4 biological

replicates for each cell line). C, D) Colony formation assay of HepG2 C), Huh6 C), and HLE D) treated with different dosages of JNKi as indicated ($n = 4$ biological replicates for each cell line). E) Cell viability assay of HepG2 treated with different dosages of ER stressors, including Thap, Tun, and CPA, as indicated ($n = 4$ biological replicates for each cell line). In B-E), results are shown as mean ± SEM. Statistical analyses using one-way ANOVA B-D). Statistical significance is indicated as $^{**}p < 0.01$, $^{***}p < 0.001$.

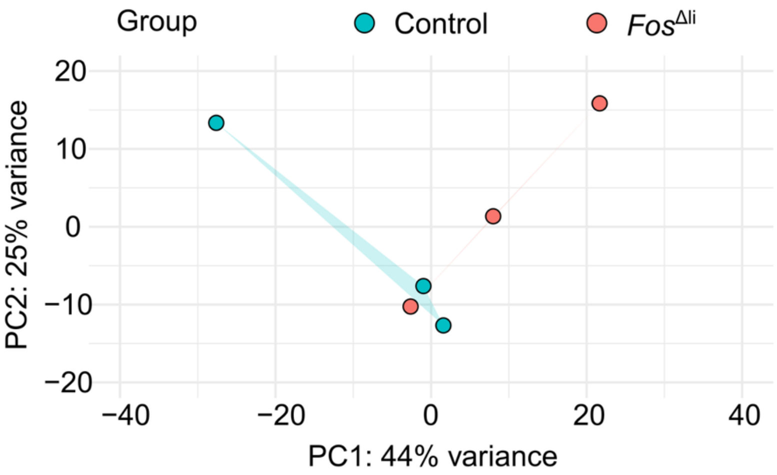

Figure S8. RNA-Seq PCA plot reveals variation between control and *Fos*$^{\Delta li}$ mice. RNA-Seq PCA plot using top 5000 variable genes depicting the variation between control and *Fos*$^{\Delta li}$ (*n* = 3 mice per group). PC1 and PC2 collectively explained 69 % of the variability among different groups, with PC1 accounting for 44 % and PC2 for 25 % of the variance, respectively.

Primary antibody	Designation	Source or reference	Cat#	Additional information
AKT	Akt (pan) (40D4) Mouse mAb	Cell Signaling Technology	2920	WB 1:2000
ALB	Albumin Antibody	Cell Signaling Technology	4929	WB 1:1000
c-Fos	Rabbit polyclonal anti-protein c-Fos	Cell Signaling Technology	4384S	WB 1:1000
c-Fos	Rabbit monoclonal anti-protein c-Fos	Cell Signaling Technology	2250S	WB 1:1000 IF 1:125
Cleaved Caspase 3	Rabbit monoclonal anti-protein cleaved caspase 3	Cell Signaling Technology	9664S	WB 1:1000
ERK	p44/42 MAPK (Erk1/2) Antibody	Cell Signaling Technology	9102	WB 1:1000
GAPDH	Rabbit polyclonal anti-glyceraldehyde-3-phoshphate dehydrogenase	R&D Systems	2275-PC-100	WB 1:5000
IR	Insulin Receptor β (4B8) Rabbit mAb	Cell Signaling Technology	3025	WB 1:1000
JNK	SAPK/JNK Antibody	Cell Signaling Technology	9252	WB 1:1000
p-AKT	Phospho-Akt (Ser473) (D9E) XP® Rabbit mAb	Cell Signaling Technology	4060	WB 1:2000
p-ERK	Phospho-p44/42 MAPK (Erk1/2) (Thr202/Tyr204) (D13.14.4E) XP® Rabbit mAb	Cell Signaling Technology	4370	WB 1:2000
p-IR	Phospho-IGF-I Receptor β (Tyr1135/1136)/Insulin Receptor β (Tyr1150/1151) (19H7) Rabbit mAb	Cell Signaling Technology	3024	WB 1:1000
p-JNK	Phospho-SAPK/JNK (Thr183/Tyr185) Antibody	Cell Signaling Technology	9251	WB 1:1000
p-p38	Phospho-p38 MAPK (Thr180/Tyr182) (D3F9) XP® Rabbit mAb	Cell Signaling Technology	4511	WB 1:1000
p38	p38 MAPK (D13E1) XP® Rabbit mAb	Cell Signaling Technology	8690	WB 1:1000
PLIN2	Guinea pig polyclonal anti-protein Perilipin 2 (N-terminus aa 6-27)	PROGEN Biotechnik	GP47	WB 1:1000
PPARγ	Rabbit monoclonal anti-PPARγ	Cell Signaling Technology	2443	WB 1:500
α-Tubulin	Mouse monoclonal anti-alpha-tubulin	Sigma-Aldrich	T5168	IF 1:500
β-actin	Anti-β-Actin antibody, Mouse monoclonal	Sigma-Aldrich	A1978	WB 1:5000

Table S1. List of primary antibodies used for western blot and immunofluorescence analysis.

Secondary antibody	Designation	Source or reference	Cat#	Additional information
Donkey anti-Mouse 555	Donkey anti-Mouse IgG (H+L) Highly Cross-Adsorbed Secondary Antibody, Alexa Fluor Plus 555	Thermo Fisher Scientific	A32773	IF 1:500
Donkey anti-Rabbit 488	Donkey anti-Rabbit IgG (H+L) Highly Cross-Adsorbed Secondary Antibody, Alexa Fluor 488	Thermo Fisher Scientific	A21206	IF 1:500
Goat anti-guinea pig	Anti-guinea pig IgG goat polyclonal, HRP conjugate	PROGEN Biotechnik	90001	WB 1:5000
Goat anti-Mouse	Goat Anti-Mouse Immunoglobulins/HRP (affinity isolated)	Dako	P0447	WB 1:5000
Goat anti-Rabbit	Goat Anti-Rabbit Immunoglobulins/HRP (affinity isolated)	Dako	P0448	WB 1:5000

Table S2. List of secondary antibodies used for western blot and immunofluorescence analysis.

siRNA	Source	Identifiers	Target sequence
siRNA Control	Qiagen	#1027281	-
Hs siRNA FOS #1	Qiagen	SI03066028	CAGCATGGAGCTGAAGACCGA
Hs siRNA FOS #2	Qiagen	SI03091844	CTCGGGCTTCAACGCAGACTA

Table S3. siRNAs used in the study to knockdown c-Fos in HCC cell lines.

Gene	qPCR F	qPCR R	Standard F	Standard R
Acaca	AGCCAGAAGGGACAGTAGAA	CTCAGCCAAGCGGATGTAAA	GCGCTTACATTGTGGATGGC	AAGCCTTCACTGTGCCTTCA
Acly	TTCGTCAAACAGCACTTCC	ATTTGGCTTCTTGGAGGTG	ACACCATCATCTGTGCTCGG	ATCCCAGGGGTGACGATACA
Cpt1α	GCTGATGACGGCTATGGTGT	AAAGCGGTGTGAGTCTGTCT	CCACAACAACGGCAGAGCA	TCAGGAGCAACACCTATTCATTTG
Fasn	CACAGTGCTCAAAGGACATGCC	CACCAGGTGTAGTGCCTTCCTC	ACTTCCTCTGGGATGTGCCT	GTCAGCACTGCTCTCGTTGA
Fos	AGCAGCTATCTCCTGAAGAGG	TCTGTCTCCGCTTGGAGTGT	CTCCTGTCAACACACAGGACTT	GTTGATCTGTCTCCGCTTGGA
Gapdh	AGTTCAACGGCACAGTCAAG	TACTCAGCACCAGCATCACC	ATGACTCTACCCACGGCAAG	TGTGAGGGAGATGCTCAGTG
Nfe212	ACTACAGTCCCAGCAGAGTGAT	TCACACACTTTCTGCGTGCT	ACTACAGTCCCAGCAGAGTGATG	AGACACTGCACTGCAACAAG
Pparα	GCTGTAAGGGCTTCTTTCGG	GCGAATTGCATTGTGTGACAT	GCATGTGAAGGCTGTAAGGGC	GACAAAAGGCGGGTTGTTGCT
Pparγ1	CCAAGAATACCAAAGTGCGATCA	AAAACCCTTGCATCCTTCACAA	GCTCCAAGAATACCAAAGTGCGA	AACCTGATGGCATTGTGAGACA
Pparγ2	TGCCTATGAGCACTTCACAAG	TCTACTTTGATCGCACTTTGGTA	AGCATGGTGCCTTCGCTGAT	GCCCAAACCTGATGGCATTGTG

Table S4. List of primers used for qPCR**.** Real-time quantitative PCR was performed using the Bio-Rad CFX96 machine (Bio-Rad Laboratories, Hercules, CA) and the SYBR green PCR Master Mix (Bio-Rad Laboratories).
